# miRNA signatures underlie chemoresistance in the gemcitabine-resistant pancreatic ductal adenocarcinoma cell line MIA PaCa-2 GR

**DOI:** 10.3389/fgene.2024.1393353

**Published:** 2024-06-11

**Authors:** Ryan N. Fuller, Paul A. Vallejos, Janviere Kabagwira, Tiantian Liu, Charles Wang, Nathan R. Wall

**Affiliations:** ^1^ Division of Biochemistry, Department of Basic Science, Center for Health Disparities and Molecular Medicine, Loma Linda, CA, United States; ^2^ Center for Genomics, Loma Linda University School of Medicine, Loma Linda, CA, United States; ^3^ Division of Microbiology, Department of Basic Science, Loma Linda University School of Medicine, Loma Linda, CA, United States; ^4^ Department of Radiation Medicine, James M. Slater, MD Proton Treatment and Research Center, Loma Linda University School of Medicine, Loma Linda, CA, United States

**Keywords:** miRNA, chemoresistance, pancreatic cancer, gemcitabine, FOLFIRINOX, biomarkers

## Abstract

**Introduction:** Chemotherapy resistance remains a significant challenge in the treatment of pancreatic adenocarcinoma (PDAC), particularly in relation to gemcitabine (Gem), a commonly used chemotherapeutic agent. MicroRNAs (miRNAs) are known to influence cancer progression and chemoresistance. This study investigates the association between miRNA expression profiles and gemcitabine resistance in PDAC.

**Methods:** The miRNA expression profiles of a gemcitabine-sensitive (GS) PDAC cell line, MIA PaCa-2, and its gemcitabine-resistant (GR) progeny, MIA PaCa-2 GR, were analyzed. miRNA sequencing (miRNA-seq) was employed to identify miRNAs expressed in these cell lines. Differential expression analysis was performed, and Ingenuity Pathway Analysis (IPA) was utilized to elucidate the biological functions of the differentially expressed miRNAs.

**Results:** A total of 1867 miRNAs were detected across both cell lines. Among these, 97 (5.2%) miRNAs showed significant differential expression between the GR and GS cell lines, with 65 (3.5%) miRNAs upregulated and 32 (1.7%) miRNAs downregulated in the GR line. The most notably altered miRNAs were implicated in key biological processes such as cell proliferation, migration, invasion, chemosensitization, alternative splicing, apoptosis, and angiogenesis. A subset of these miRNAs was further analyzed in patient samples to identify potential markers for recurrent tumors.

**Discussion:** The differential miRNA expression profiles identified in this study highlight the complex regulatory roles of miRNAs in gemcitabine resistance in PDAC. These findings suggest potential targets for improving prognosis and tailoring treatment strategies in PDAC patients, particularly those showing resistance to gemcitabine. Future research should focus on validating these miRNAs as biomarkers for resistance and exploring their therapeutic potential in overcoming chemoresistance.

## Introduction

Pancreatic ductal adenocarcinoma (PDAC) is one of the most lethal forms of cancer in both men and women. PDAC is currently ranked the third leading cause of cancer death in the United States for men and fourth for women. Nearly 64,000 patients are expected to be diagnosed with PDAC in 2023, with nearly 50,500 patients dying of PDAC this year alone (American Cancer Society and I, 2023). PDAC is ranked second for the lowest 5-year survival rates of any cancer (11%), with most patients passing away within 1 year of diagnosis. PDAC is becoming more common and is projected to be the second leading cause of cancer death in the US by the year 2030 (Rahib et al., 2014), with chemoresistance playing a primary role in this poor prognosis.

Over the past decade, PDAC survival has improved only marginally, with few novel clinical interventions to significantly improve patient life. With the advent of gemcitabine (Gem) treatment, the median overall survival rate for PDAC improved over 5-FU monotherapy from 4.41 to 5.65 months, and the 1-year survival rate improved from 2% to 18% (Burris et al., 1997). FOLFIRINOX, a combination of folinic acid (leucovorin) 5-fluorouracil (5-FU), irinotecan, and oxaliplatin, is a novel therapeutic regimen for metastatic pancreatic cancer that has shown superiority to Gem by improving median survival rates from 6.8 months to 11.1 months (Conroy et al., 2011). Although FOLFIRINOX improves the patient survival rate by 5 months, it has not reduced the rate at which this cancer kills. This study was conducted to address this concern and provide a new framework to attack pathways pertinent to the onset of PDAC chemoresistance.

Next-generation sequencing (NGS) is a powerful tool for identifying biological responses to therapy. Informatic software and data availability have significantly improved the ability to identify key pathways for resistance and to propose novel therapeutic interventions in the form of regulatory genetic sequences. The most well-studied form of regulatory genetic sequences in our body is miRNA. This classification of small non-coding RNAs regulates translation by inhibiting mRNA from binding to the ribosome through binding to mRNA 3′UTR regions. miRNAs are ∼18–22 nts in length and contain AGO2-binding regions and a seed region, which guides the AGO2 catalytic activity to specific homologous sequences on mRNA 3′UTRs. These seed sequences range from 6 nts to 8 nts in length and may have multiple binding sites on the same 3′UTR region. Advanced software allows for the prediction of regulatory efficiency of miRNAs based on well-documented and conserved 3′UTR regions of mRNAs. An ever-increasing body of literature supports the use of miRNAs as biomarkers for disease presence, initiators of drug resistance, and even as therapeutic agents themselves (Hossen et al., 2022; Lahoz et al., 2022).

To this end, we generated a Gem-resistant cell line, MIA PaCa-2 GR, and performed miRNA-seq utilizing Illumina NextSeq 550, a next-generation sequencing platform, on Gem-resistant MIA-PaCa-2 cells (MP2 GR) and Gem-sensitive MIA-PaCa-2 cells (MP2 GS). We have characterized a novel miRNA profile between sensitive and highly resistant MIA-PaCa-2 cell lines to identify regulatory miRNAs that play a role in chemoresistance-promoting pathways. We have further utilized analytical software to predict the miRNA interactions with key proteins responsible for Gem resistance. In the end, we identified 97 differentially expressed miRNAs in this profile and found several miRNAs that are involved in chemoresistance through the regulation of survivin, a potent inhibitor of apoptosis.

## Materials and methods

### Cell culture

The PDAC cell line MIA PaCa-2 was acquired from ATCC and maintained in DMEM (Mediatech, Manassas, VA) supplemented with 2.5% horse serum, 100 units of penicillin, 100 μg/mL of streptomycin, 300 µ/mL of L-glutamine, and 10% USDA-sourced heat-inactivated fetal bovine serum (Mediatech, Manassas, VA). MIA PaCa-2 GR was generated as previously described (Fuller et al., 2022) and maintained in the same media used to culture MIA PaCa-2 and further supplemented with 500 nM of gemcitabine hydrochloride (Sigma-Aldrich, St Louis, MO). In all the experiments, cells were cultured at 37°C in a humidified atmosphere containing 5% CO_2_ to 70%–80% confluency prior to use.

### RNA isolation

Total RNA was isolated using the TRIzol reagent (Invitrogen, Carlsbad, CA) according to the manufacturer’s instructions. Briefly, harvested cells were lysed in 500 µL TRIzol reagent with the pellet frozen at −80°C. Samples were thawed at room temperature, after which 100 µL of chloroform was added, and cells were incubated at room temperature. Samples were shaken vigorously for 15 s and then allowed to stand for 15 min at room temperature prior to centrifuging for 15 min at 12,000 x g at 4°C. The aqueous phase was transferred to a fresh tube, and 250 μL of 2-propanol was added, followed by an additional 10-min centrifugation. RNA pellets were washed in cold ethanol, and the concentration was determined by measuring the absorbance at 260/280 nm on a NanoDrop spectrophotometer (Thermo Scientific, Waltham, MA).

### miRNA-seq library construction and sequencing

miRNA-seq libraries were constructed using the QIAseq miRNA library kit (QIAGEN, Germantown, MD) following the manufacturer’s instructions. miRNAs were processed as previously recorded (Liu et al., 2023). Briefly, 3′ and 5′ adapters, along with unique molecular identifiers, were added to small RNAs. Reverse transcription was performed to convert the miRNAs into cDNAs, followed by a 22-cycle PCR amplification. An assigned index was given to each sample for the multiplexing. The amplified fragments were double size selected by using QIAseq magnetic beads for fragments of DNA with 150–200 bp. Libraries were quantified by the Qubit 3.0 HS dsDNA assay (Thermal Fisher Scientific, Waltham, MA). Library size and quality were examined using the TapeStation 2200 (Agilent, Santa Clara, CA). miRNA-seq libraries were sequenced at 76-bp single-end on an Illumina NextSeq 550 at the Center for Genomics, LLU, with a final loading concentration of 2.1 p.m.

### miRNA identification and differential miRNA expression analysis

miRNA data were processed as previously recorded (Liu et al., 2023). All sequencing data were demultiplexed and converted to fastq files using bcl2fastq (Illumina Inc., San Diego, CA). After quality control with the FastQC tool, fastq files were processed using the QIAseq miRNA quantification feature provided by QIAGEN’s GeneGlobe Data Analysis Center (Qiagen, Germantown, MD). Adapters from read sequences were trimmed, and low-quality bps were removed by Cutadapt (Martin, 2011). Reads with fewer than 16 bps and 10 assigned index counts were excluded from the analysis. Reads were aligned to miRbase (v21) using Bowtie with no more than two mismatches. Reads with more than 20 assigned index counts were used for differential expression analysis by the R/Bioconductor (Gentleman et al., 2004) software package, DESeq2 (Hanahan and Weinberg, 2011). Differentially expressed miRNAs (DEmiR) with false discovery rate (FDR)-adjusted *p*-values (q-values) of less than 0.01 and logarithmic 2-fold changes of 0.5 were selected. Sample distance heatmaps were generated using the R package pheatmap (Kolde R. 2019).

### Validation by RT-qPCR on key miRNAs

The top three overexpressed miRNAs in the GR cell line, miR-205-5p, miR-34a-3p, and miR-3529-5p, as well as an internal control recommended by ThermoFisher, miR-26a-3p, were used to validate this dataset. miRNAs were isolated using mirVana™ (Invitrogen, Carlsbad, CA) and processed using 50 ng of RNA sample along with a TaqMan™ Advanced miRNA cDNA Synthesis Kit (Applied Biosciences, Beverly Hills) to produce cDNA templates of key miRNAs. TaqMan™ Fast Advanced Master Mix for qPCR was used along with the TaqMan™ Advanced miRNA Assays (Applied Biosciences, Beverly Hills) for miR-205-5p, miR-34a-3p, and miR-3529-5p. All samples were prepared according to manufacturer-recommended protocols. Sample CT values were normalized by taking the difference of CTs between the MP2 and MP2GR cells of miR-26a-3p and subtracting the result from the CTs collected from the sample.

### Pathway analysis

MiRNA targets were predicted through the use of QIAGEN Ingenuity Pathway Analysis (IPA, QIAGEN Inc. https://www.qiagenbioinformatics.com/products/ingenuity-pathway-analysis). DEmiRs were imported into the IPA miRNA target filter, and target interactions with moderate or high confidence and experimentally observed interactions were selected for analysis. Gene ontology (GO) enrichment was performed using built-in IPA tools with DEmiRs.

### miEAA analysis

miRNA enrichment analysis and annotation (miEAA) was used to develop gene ontology for the miRNA profile (Aparicio-Puerta et al., 2023). Upregulated and downregulated miRNAs were loaded together into miEAA software and were indexed by the Wald statistic generated from DESeq2. Significant pathways were determined by miEAA software through https://ccb-compute2.cs.uni-saarland.de/mieaa/ with an RNADisease repository, miRWalk V2.0, and GO annotations. Pathways with *p*-adjusted values < 0.05 were considered significant. Upset plots were built using UpSetR (Conway et al., 2017) in R. Ggvenn (https://github.com/yanlinlin82/ggvenn) was used to generate Venn diagrams based on miEAA data (GitHub, Inc.). Annotations from RNADisease v4.0 (Chen et al., 2023) were used to generate sets of miRNAs related to pancreatic cancer (keywords: “pancreatic adenocarcinoma,” “pancreatic cancer,” “pancreatic ductal adenocarcinoma,” and “pancreatic carcinoma”) and risk factors associated with pancreatic cancer (keywords: “nicotine consumption/nicotine addiction,” “diabetes,” “obesity,” “pancreatitis,” “acute pancreatitis,” “chronic pancreatitis,” and “metabolic syndrome”). The intersection between the DEmiRs generated from GS and GR cells, miRNAs annotated for risk factors for PDAC, and miRNAs annotated for PDAC (15 miRNAs) were used for further analysis in patient samples.

### GeneHancer and UniProt associations

Data from the GeneHancer database (Fishilevich et al., 2017) utilizing Gene Cards (https://www.genecards.org/) were searched for *birc5* (*surviving*) promoter and enhancer regions. All proteins with binding sites on regions scored for *birc5* expression interactions were considered (552 proteins in total). Duplicate proteins were removed from the dataset before comparison. Member protein families from the UniProt database were included with tags matching: “Polypyrimidine track binding” (39), “hnRNP” (131), “snRNP” (175), or “serine/arginine-rich splicing factor” (60). The criteria for selection were 1) human proteins, 2) ability to be visualized at the protein level, and 3) an annotation score of 5. All data lists will be made available upon request.

These five protein lists were uploaded to the IPA software to determine interactions between the DEmiR profile and protein lists. Interactions were filtered in IPA for only human interactions that are highly predicted or experimentally observed by the TargetScan Human (v.7) context scoring system (Agarwal et al., 2015). Data were compiled and visualized by a circos plot (Krzywinski et al., 2009) using a plugin from Galaxy (https://usegalaxy.org/). Full lists of proteins and interactions will be made available upon request.

### TCGA analysis

Patient data were analyzed from data generated by The Cancer Genome Atlas (TCGA) Research Network: https://www.cancer.gov/tcga, utilizing the PAAD and CPTAC3 datasets that contain miRNA data on patient tumor samples. Expression data taken from both patient datasets were analyzed using the available BAM files. Briefly, BAM files were analyzed through feature counts (Liao et al., 2013) with alignment to the genome assembly GRCh38. miRNA accessions from this were converted to precursor miRNAs using the miRbase converter tool (Xu et al., 2018). For comparison with the differentially expressed miRNAs from cell lines, all miRNAs were converted to the precursors for use in prediction analysis. Logical regression modeling was used in R to establish the sensitivity and specificity of miRNAs to differentiate between groups. Data were plotted into a receiver operating characteristic (ROC) curve for visualization and to find the c-statistic/area under the curve (AUC) (Grund and Sabin, 2010). For the TCGA-PAAD dataset, patients were separated into two groups based on whether they received chemotherapeutic treatment prior to tumor sampling. The CPTAC3 patient set was separated into two groups based on recurrence or non-recurrence upon data acquisition. Groups were established based on available metadata. ROC curves were used to identify the predictive capacity of these groups in the respective datasets.

### Statistics

Experiments were performed using at least three biological replicates. The differences between groups were analyzed by independent Student t-tests, while the differences between multiple groups were compared using Welch-ANOVA. *p*-values *p < 0.05, **p < 0.01, and ***p < 0.005 were considered statistically significant. ROC curves were illustrated by logical regression modeling, with the area under the curve representing the overall predictive capacity/C-statistic threshold.

## Results

### Expression profile of miRNAs in the GR cell line

**FIGURE 1:**
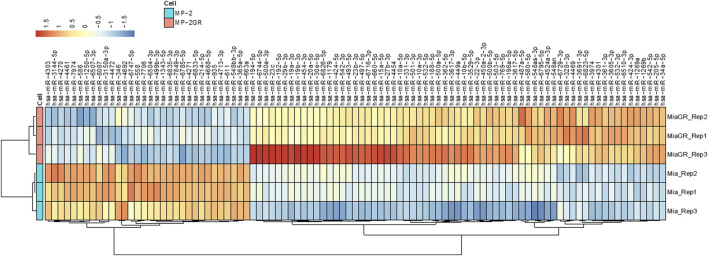
Hierarchical cluster of differentially expressed miRNAs in MIA PaCa-2 parental (MP-2) and MIA PaCa-2 GR (MP-2GR) cell lines. The heatmap shows differentially expressed miRNAs within a hierarchical cluster from miRNAs in Table 1. Vertical columns represent different miRNAs, and horizontal rows represent different samples. The color scale illustrates the relative expression levels of miRNAs. Red indicates high relative expression levels of miRNAs, and blue indicates low relative expression levels of miRNAs (miR) between GR and GS cells.

The development and characterization of MIA PaCa-2 GR cells from their isogenic parental MIA PaCa-2 GS has recently been described by our group (Fuller et al., 2022). We performed gene expression profiling in these two cell lines with the goal of characterizing transcriptional alterations upon the development of resistance to Gem. Illumina NextSeq 550 was used to sequence enriched small non-noncoding RNAs. The QIAseq miRNA library (QIAGEN, Germantown, MD) was used to align small RNA findings and was able to identify 1867 miRNAs within this sample set. An miRNA expression profile between the two cell lines was determined by DESeq2 (Love et al., 2014) with a *p*-value cutoff of *p* ≤ 0.05 between the resistant and isogenic-sensitive cell lines. A total of 97 (5.2%) miRNAs had significant differential expression (q < 0.05) between the GR and parental MIA PaCa-2 cells. Of these miRNAs, 65 (3.5%) were upregulated, and 32 (1.7%) miRNAs were downregulated (Table 1). Hierarchical clustering based on differentially expressed RNA transcripts revealed a distinct transcriptomic profile of these differentially expressed miRNAs, which is shown as a hierarchical clustered heat map in Figure 1.

**TABLE 1:** Complete list of DEmiRs from NGS.

Upregulated						Downregulated		
miRNA	log2 FC	FDR-adjusted *p*-value	miRNA	log2 FC	FDR-adjusted *p*-value	miRNA	log2 FC	FDR-adjusted *p*-value
hsa-miR-205-5p	6.452906395	1.83E−29	hsa-miR-30e-5p	2.43295747	2.47E−02	hsa-miR-4271	−3.428385829	1.96E−08
hsa-miR-34a-5p	5.813896088	1.83E−29	hsa-miR-454-3p	2.410392865	4.32E−02	hsa-miR-7849-3p	−3.275769274	3.06E-10
hsa-miR-6744-5p	5.040554843	9.87E−03	hsa-miR-23b-3p	2.282158683	3.08E−03	hsa-miR-6835-3p	−3.168711639	2.15E−05
hsa-miR-3529-5p	4.798954296	4.22E−04	hsa-miR-20a-5p	2.238637482	3.97E−02	hsa-miR-663a	−2.982471138	1.32E−09
hsa-miR-10a-5p	4.142987006	4.02E−07	hsa-miR-3605-3p	2.219331234	1.72E−04	hsa-miR-6833-5p	−2.773353496	2.53E−05
hsa-miR-3617-5p	4.100460927	2.79E−04	hsa-miR-1269a	2.170849703	1.58E−04	hsa-miR-4303	−2.738894303	2.18E−03
hsa-miR-493-5p	4.084720541	1.82E−02	hsa-miR-532-5p	2.167276755	9.66E−05	hsa-miR-1343-5p	−2.708305835	2.08E−05
hsa-miR-4524a-3p	3.915829109	5.78E−04	hsa-miR-196a-5p	2.123504892	5.96E−04	hsa-miR-3150a-3p	−2.706641591	1.01E−03
hsa-miR-424-5p	3.886841391	1.74E−02	hsa-miR-3691-3p	2.111900883	2.15E−02	hsa-miR-346	−2.421623531	3.72E−02
hsa-miR-10a-3p	3.866951212	3.55E−10	hsa-miR-6716-3p	2.108916616	1.86E−02	hsa-miR-935	−2.384057289	1.26E−06
hsa-miR-10b-5p	3.652452388	1.61E−02	hsa-miR-4301	1.980371335	2.49E−04	hsa-miR-7974	−2.308632389	2.49E−04
hsa-miR-487a-5p	3.526923868	1.49E−02	hsa-miR-23c	1.970670515	4.80E−02	hsa-miR-4692	−2.28091657	3.64E−02
hsa-miR-19a-3p	3.492479139	4.35E−02	hsa-miR-424-3p	1.905917892	1.73E−03	hsa-miR-558	−2.258266932	4.92E−03
hsa-miR-6826-5p	3.433654095	3.67E−02	hsa-miR-6823-5p	1.88539802	1.02E−02	hsa-miR-4713-3p	−2.205438564	6.96E−04
hsa-miR-542-3p	3.37805884	2.25E−02	hsa-miR-27b-3p	1.86130327	3.72E−02	hsa-miR-4668-5p	−2.192894276	1.51E−04
hsa-miR-660-5p	3.252069193	2.17E−03	hsa-miR-3974	1.852045508	2.17E−03	hsa-miR-548bb-3p	−2.17527534	1.71E−02
hsa-miR-548an	3.240904108	1.44E−02	hsa-miR-188-5p	1.834484985	1.24E−02	hsa-miR-6504-3p	−2.13638109	1.59E−02
hsa-miR-1179	3.221333845	4.80E−02	hsa-miR-500a-3p	1.768875286	4.07E−02	hsa-miR-214-5p	−2.129236273	8.33E−03
hsa-miR-449a	3.208429903	1.86E−02	hsa-miR-362-5p	1.754063378	3.69E−02	hsa-miR-6507-3p	−1.978088994	1.23E−02
hsa-miR-450a-2-3p	3.169177772	1.16E−02	hsa-miR-22-3p	1.753743048	1.35E−02	hsa-miR-8087	−1.911079912	2.56E−02
hsa-miR-4469	3.144768651	2.15E−05	hsa-miR-501-3p	1.70841929	2.18E−03	hsa-miR-611	−1.903992446	4.32E−02
hsa-miR-6795-5p	3.137450672	8.02E−03	hsa-miR-500a-5p	1.708298323	1.24E−02	hsa-miR-3144-5p	−1.88508858	2.25E−02
hsa-miR-580-5p	3.137427436	4.32E−02	hsa-miR-542-5p	1.702633782	1.02E−02	hsa-miR-1250-5p	−1.851497419	2.29E−02
hsa-miR-3617-3p	2.986847336	3.24E−02	hsa-miR-4454	1.690411106	4.32E−02	hsa-miR-586	−1.833196701	2.13E−02
hsa-miR-192-5p	2.844225121	1.02E−02	hsa-miR-497-5p	1.683713826	3.86E−02	hsa-miR-4461	−1.832783235	9.90E−03
hsa-miR-548as-3p	2.626273501	4.07E−02	hsa-miR-331-3p	1.62175797	9.81E−03	hsa-miR-499b-3p	−1.829204548	4.12E−03
hsa-miR-6510-3p	2.612605323	1.96E−06	hsa-miR-500b-5p	1.482412496	1.50E−02	hsa-miR-6747-5p	−1.802985512	4.01E−02
hsa-miR-15a-5p	2.596530688	1.23E−02	hsa-miR-328-3p	1.466445165	2.25E−02	hsa-miR-4270	−1.790135215	1.24E−02
hsa-miR-29b-3p	2.581217379	1.57E−02	hsa-miR-532-3p	1.439545205	2.13E−03	hsa-miR-4472	−1.625164285	1.44E−02
hsa-miR-6729-3p	2.57873501	1.44E−02	hsa-miR-769-5p	1.312120797	2.02E−02	hsa-miR-7706	−1.61261424	1.76E−02
hsa-miR-1269b	2.534821602	4.50E−09	hsa-miR-503-5p	1.299242426	1.44E−02	hsa-miR-6785-5p	−1.490229486	3.11E−02
hsa-miR-19b-3p	2.471155028	4.07E−02	hsa-miR-361-3p	1.053585418	3.72E−02	hsa-miR-365a-5p	−1.473978483	4.84E−02
hsa-miR-194-5p	2.443167865	3.30E-02						

miRNA are sorted by log2 (fold change) and are separated by upregulated (left) and downregulated (right). The FDR-adjusted *p*-value represents the *p*-value adjusted by the B-H false discovery rate through the DESeq2 R package. Factors represented were filtered to include only significant miRNAs with a *p*-value < 0.05 and LFC > 0.5.

**FIGURE 2:**
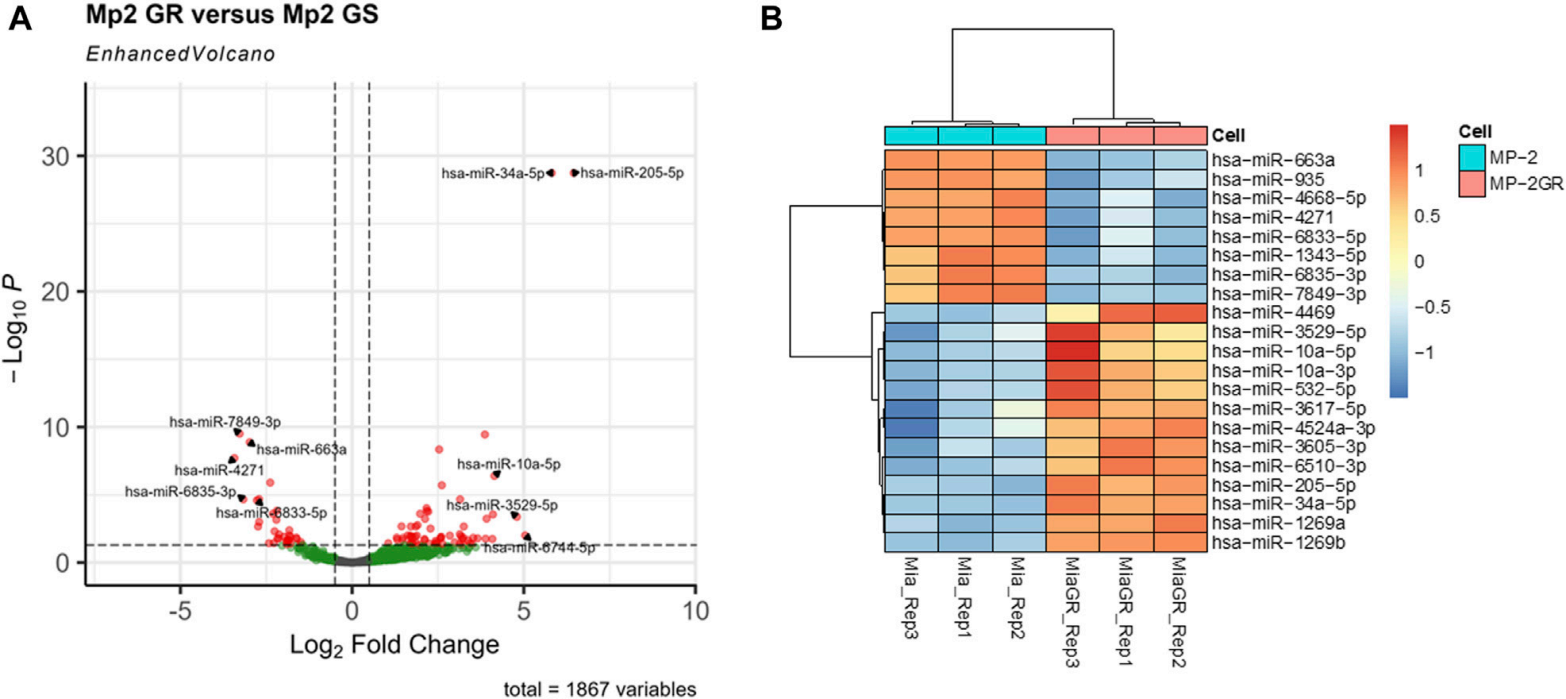
Volcano plot and hierarchical cluster of differentially expressed miRNAs in MIA PaCa-2 parental (MP-2) and MIA PaCa-2 GR (MP-2GR) cell lines. **(A)** The volcano plot has the top five upregulated and downregulated factors by log-fold change (LFC) highlighted, as well as the cutoff points used for this study. Vertical dashed lines represent an LFC cutoff of 0.05, and horizontal dashed lines represent a *p*-adj cutoff of 0.05. Gray points did not meet either cutoff, green points met the LFC cutoff but not the significance cutoff, and red points met both criteria. **(B)** The heatmap shows differentially expressed miRNA within a hierarchical cluster of miRNAs from Table 2. Rows represent different miRNAs, and columns represent different samples. The color scale illustrates the relative expression levels of miRNAs. Red indicates high relative expression levels of miRNAs, and blue indicates low relative expression levels of miRNAs (miR) between GR and GS cells.

Furthermore, stringency in this dataset was enhanced upon increasing the log fold change (LFC) threshold. Figure 2A shows a volcano plot of significant miRNAs. The top five upregulated (miR-205-5p, miR-34a-3p, miR-3529-5p, miR-6744-5p, and miR-10a-5p) and downregulated miRNAs (miR-4271, miR-7849-3p, miR-6835-3p, miR-663a, and miR-6833-5p) with the greatest differential LFC among significant miRNAs are annotated. Figure 2B represents a hierarchical clustering heat map with miRNAs that surpass expression LFC > 2.0 (|log_2_FC| ≥ 2.0) with a q-value cutoff of ≤ 0.01 between the GS and GR cell lines. These results indicated 21 miRNAs with significant differences between the GR and GS cells, of which 13 were upregulated, and eight were downregulated (Table 2). While a less sensitive metric compared to miRNA-seq, data were validated by RT-qPCR (Supplemental Figure S1) to indicate expression differentials between GR and GS cells.

**TABLE 2:** A refined list of miRNA factors from the total.

Upregulated			Downregulated		
miRNA	log2 FC	FDR-adjusted *p*-value	miRNA	log2 FC	FDR-adjusted *p*-value
hsa-miR-205-5p	6.452906395	6.47E−25	hsa-miR-4271	−3.428385829	4.58E−06
hsa-miR-34a-5p	5.813896088	1.25E−24	hsa-miR-7849-3p	−3.275769274	2.35E−07
hsa-miR-3529-5p	4.798954296	2.92E−03	hsa-miR-6835-3p	−3.168711639	8.54E−04
hsa-miR-10a-5p	4.142987006	1.58E−05	hsa-miR-663a	−2.982471138	1.44E−06
hsa-miR-3617-5p	4.100460927	2.92E−03	hsa-miR-6833-5p	−2.773353496	1.40E−03
hsa-miR-4524a-3p	3.915829109	5.60E−03	hsa-miR-1343-5p	−2.708305835	1.24E−03
hsa-miR-10a-3p	3.866951212	1.53E−07	hsa-miR-935	−2.384057289	4.23E−04
hsa-miR-4469	3.144768651	8.54E−04	hsa-miR-4668-5p	−2.192894276	9.46E−03
hsa-miR-6510-3p	2.612605323	4.10E−04			
hsa-miR-1269b	2.534821602	8.36E−06			
hsa-miR-3605-3p	2.219331234	9.51E−03			
hsa-miR-1269a	2.170849703	9.51E−03			
hsa-miR-532-5p	2.167276755	7.69E−03			

This represents the 21 top up- and downregulated miRNAs when filtering the dataset by LFC > 2 and *p* adj > 0.001.

### Ingenuity pathway analyses of differentially expressed miRNAs

**FIGURE 3:**
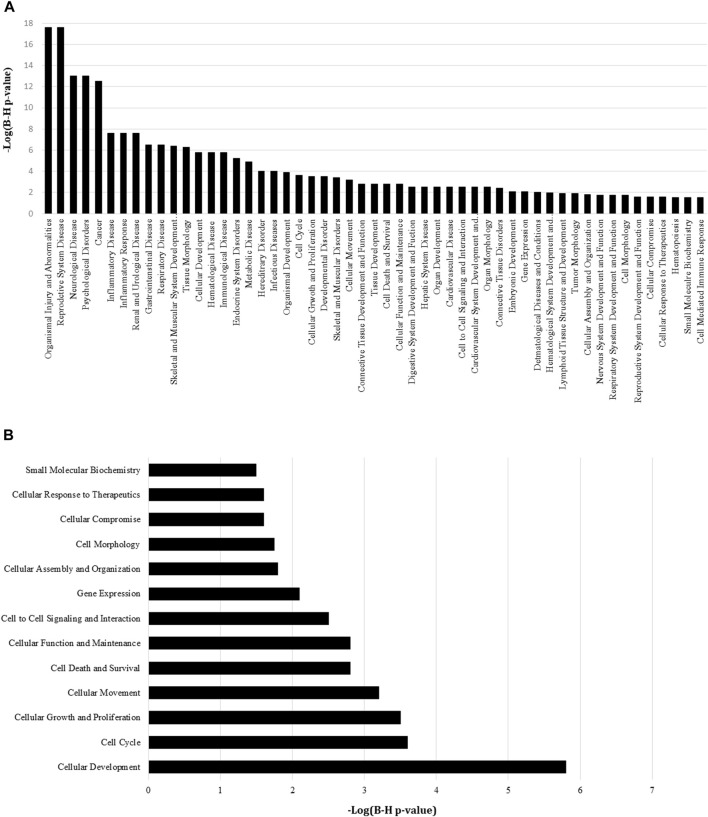
IPA pathway analysis of differentially expressed miRNAs between the MIA PaCa-2 GR and MIA PaCa-2 parental cell line. **(A)** A higher −log (B–H *p*-value) shown on the left *Y*-axis represents more significant pathways with a cutoff threshold set in the IPA software at ≥ 1.3. **(B)** A higher −log (B–H *p*-value) shown on the bottom *X*-axis represents more significant pathways with a cutoff threshold set in the IPA software at ≥ 1.3.

miRNA targets were predicted using IPA to find miRNA pathway interactions and infer potential mechanistic impact from this dataset. A core analysis of the DEmiRs (97 miRNAs differentially expressed with *p*-value > 0.05) was conducted, and protein annotations were grouped to create gene ontology reports. Annotated pathways were considered significant by Benjamini–Hochberg adjusted *p*-value < 0.05 (−log (B-H *p*-value) ≥ 1.3). Using protein target disease annotations from IPA, the most significantly represented diseases associated with these miRNAs were organismal injury and abnormalities, reproductive system disease, neurological disease, psychological disorders, and cancer. Additionally, we observed significant DEmiR enrichment in pathways involved in PDAC progression, including those associated with inflammatory disease and response, a number of anatomical system pathologies, development, cellular function and control, and gene expression (Figure 3A). Furthermore, we performed pathway analysis using IPA’s cellular functions and pathway annotations. Here, we observed a significant enrichment in the canonical pathways associated mainly with cellular development, proliferation, gene expression, small molecular biochemistry, and most notably, cell death/survival and cellular response to therapeutics (Figure 3B).

Many interesting pathways where these miRNA target proteins infer a potential mechanism of the underlying chemoresistance differences observed between the GR and GS cells were identified. Among IPA’s preconstructed pathways, the 14-3-3-mediated pathway, cancer drug resistance pathway, AMPK signaling pathway, adipogenesis pathway, 3-phosphoinositide biosynthesis pathway, aryl hydrocarbon receptor signaling pathway, 4-1BB signaling in T lymphocytes, and acute myeloid leukemia signaling pathway were all found to be significantly different between the two cell lines and potentially involved in Gem resistance (Table 3).

**TABLE 3:** Pathways-specific associated differentially expressed miRNAs in the MIA PaCa-2 PDAC cell lines.

Pathway	Number of miRNAs	Directional change and fold change (log2FC)				Number of targets
14-3-3-Mediated signaling	15 miRNAs	8 up	2.167 to 6.453	7 down	−3.428 to −2.193	23 targets
Cancer drug resistance pathway	6 miRNAs	3 up	2.535 to 4.799	3 down	−3.428 to −2.193	5 targets
AMPK signaling	4 miRNAs	2 up	2.167 to 5.814	2 down	−3.428 to −2.708	3 targets
Adipogenesis pathway	5 miRNAs	3 up	2.535 to 5.814	2 down	−3.428 to −2.982	1 target
3-Phosphoinositide biosynthesis	4 miRNAs	3 up	2.535 to 6.453	1 down	−2.773	1 target
Aryl hydrocarbon receptor signaling	1 miRNA	1 up	4.143			1 target
4-1BB signaling in T lymphocytes	1 miRNA	1 up	2.167			1 target
Acute myeloid leukemia signaling	1 miRNA			1 down	−2.982	1 target

The IPA pathway highlights the different miRNA associations in the cancer drug resistance pathway. These pathways were chosen to indicate functional interactions within IPA annotations.

### Analyzing key pathways by miEAA

**FIGURE 4:**
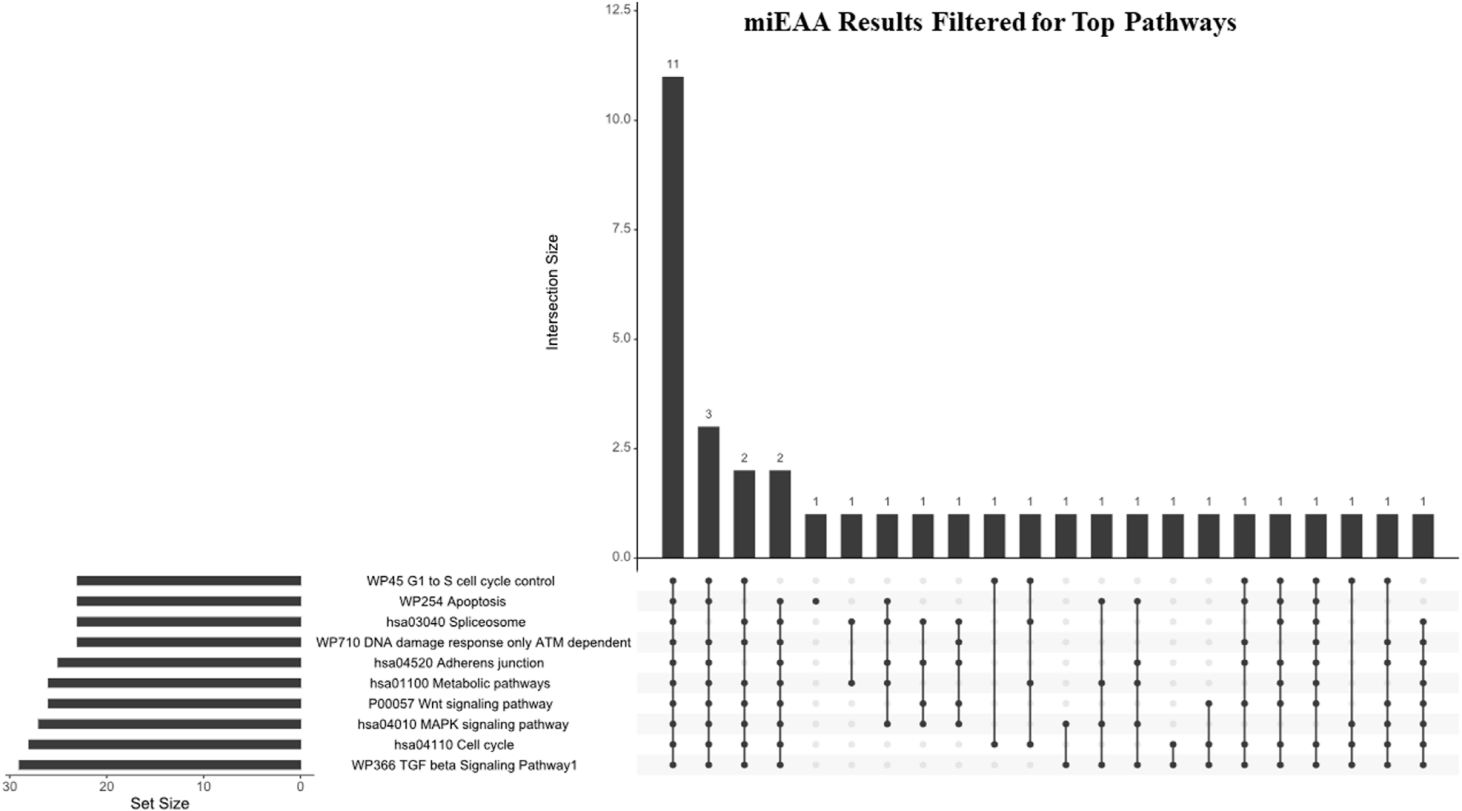
miEAA results filtered for hallmark pathways. Pathways generated from the miEAA report were screened for involvement in the 10 hallmarks of cancer. Only the top 10 enriched pathways represented by > 2 miRNA interactions and a B-H adjusted *p*-value were selected for analysis. Bars indicate the number of miRNAs shared across the connected dots, as seen below. The set size represents the total number of miRNAs from our dataset that are involved in the noted pathway.

While IPA is an accurate and powerful bioinformatics software for exploring miRNA targets, we sought to validate these findings through the use of miRNA enrichment analysis and annotation (miEAA). miEAA adds a more robust set of annotations to miRNAs from their predicted and experimentally observed targets to find pathways and diseases where these miRNAs may interact. Using the latest version of miEAA, we loaded DEmiRs and conducted gene set enrichment analysis (GSEA) indexing by the Wald statistic generated in DESeq2 and considered annotations from the RNADisease repository, miRWalk, and GO for pathway reports. Pathways with FDR-adjusted *p*-values < 0.05 were considered significant. From this annotation report, we highlighted the pathways related to the 10 hallmarks of cancer (Hanahan and Weinberg, 2011) (Table 4). In addition to filtering the dataset, we compared miRNA lists found within these pathways using an upset plot (Figure 4). The results indicate large overlaps between many key pathways in pancreatic cancer.

**TABLE 4:** miEAA miRWalk pathways: Highlighted annotations for pancreatic cancer. Annotations represent pathway names.

Annotation	Enrichment	*p*-value	Padj-value	miRNAs
WP366 TGF beta signaling pathway1	Enriched	8.62E−04	0.0152802	29
hsa04110 Cell cycle	Enriched	0.001434	0.0161662	28
hsa04010 MAPK signaling pathway	Enriched	0.002242	0.019038	27
P00057 Wnt signaling pathway	Enriched	5.98E−04	0.0152802	26
hsa01100 Metabolic pathways	Enriched	0.003366	0.0213811	26
hsa04520 Adherens junction	Enriched	0.005053	0.0256064	25
WP710 DNA damage response only ATM-dependent	Enriched	3.37E−04	0.0152802	23
hsa03040 Spliceosome	Enriched	0.001042	0.0152802	23
WP254 apoptosis	Enriched	0.011125	0.0407917	23
WP45 G1 to S cell cycle control	Enriched	0.011125	0.0407917	23
P00005 Angiogenesis	Enriched	6.26E−04	0.0152802	22
WP615 Senescence and autophagy	Enriched	5.15E−04	0.0152802	22
WP411 mRNA processing	Enriched	0.001482	0.0163021	22
WP707 DNA damage response	Enriched	0.003307	0.0213811	22
P00031 Inflammation mediated by chemokine and cytokine signaling pathway	Enriched	0.015939	0.0457874	22
hsa04630 Jak STAT signaling pathway	Enriched	0.015149	0.0457874	21
hsa04910 Insulin signaling pathway	Enriched	0.005018	0.0256064	20
hsa04115 p53 signaling pathway	Enriched	0.010694	0.0405305	19
P04393 Ras Pathway	Enriched	0.001013	0.0152802	17
P00012 Cadherin signaling pathway	Enriched	0.005128	0.0256983	16
P00013 Cell cycle	Enriched	0.005525	0.0257761	16
WP466 DNA Replication	Enriched	0.003778	0.0215702	13
hsa04340 Hedgehog signaling pathway	Enriched	0.002849	0.0213811	8
WP391 Mitochondrial gene expression	Enriched	0.013185	0.0440176	7
P02775 Salvage pyrimidine ribonucleotides	Enriched	0.0156	0.0457874	3

Only enriched pathways were selected. miEAA report generated ∼180 pathways from the DEmiRs indexed by the Wald statistic. Only enriched pathways represented by > 2 miRNA interactions and a B-H adjusted *p*-value were selected for analysis. The pathways shown were screened by involvement in cancer hallmarks from the original list (the complete, unfiltered list of miEAA findings is available in the Supplementary Figures).

The top 10 pathways generated from this report (Figure 4) were investigated next: WP336 TGF beta signaling pathway 1, hsa04110 cell cycle, hsa04010 MAPK signaling pathway, P00057 Wnt signaling pathway, hsa01100 metabolic pathways, hsa4520 adherens junction, WP710 DNA damage response only ATM-dependent, hsa03040 spliceosome, WP254 apoptosis, and WP45 G1 to S cell cycle control. Interestingly, these pathways shared 11 DEmiRs: hsa-miR-34a-5p, hsa-miR-10a-5p, hsa-miR-196a-5p, 23b-3p, hsa-miR-331-3p, hsa-miR-192-5p, hsa-miR-15a-5p, hsa-miR-22-3p, hsa-miR-10b-5p, hsa-miR-20a-5p, and hsa-miR-19b-3p. These miRNAs represent a total of between 37.9% and 47.8% of pathway set sizes. Perhaps most interesting is the representation of both antiapoptotic pathways and alternative splicing, as these pathways shared 16 DEmiRs (69.6% of their set sizes), which indicates a substantial overlap between apoptotic signaling and spliceosome interactions within these DEmiRs. This coincides with our previous reports on pancreatic cancer chemoresistance through the alternative splicing of survivin (Fuller et al., 2022).

### Building protein sets by UniProt and GeneHancer to predict DEmiR functionality

Datasets were constructed of key factors found within alternative splicing and survivin promotion to specifically explore the interactions with this miRNA profile and survivin alternative splicing. The GeneHancer database provided through UniProt indicates several significant promoter and enhancer regions for survivin. These regions are embedded with transcription factor binding sites. The factors with binding sites on significant promoter and enhancer regions were put into a list for target prediction in IPA. Additionally, UniProt was used to construct datasets from four families of alternative splicing regulators: hnRNPS, polypyrimidine tract binding proteins (PPTB family), snRNPs, and serine-rich splicing factors (SR family). Using IPA and the integrated Targetscan (v7) tool for target prediction, we considered miRNA interactions in humans with high confidence or experimentally observed predictions. Target prediction datasets were constructed for each splicing family and all the transcription factors with binding sites on survivin’s promoter and enhancer regions.

**FIGURE 5:**
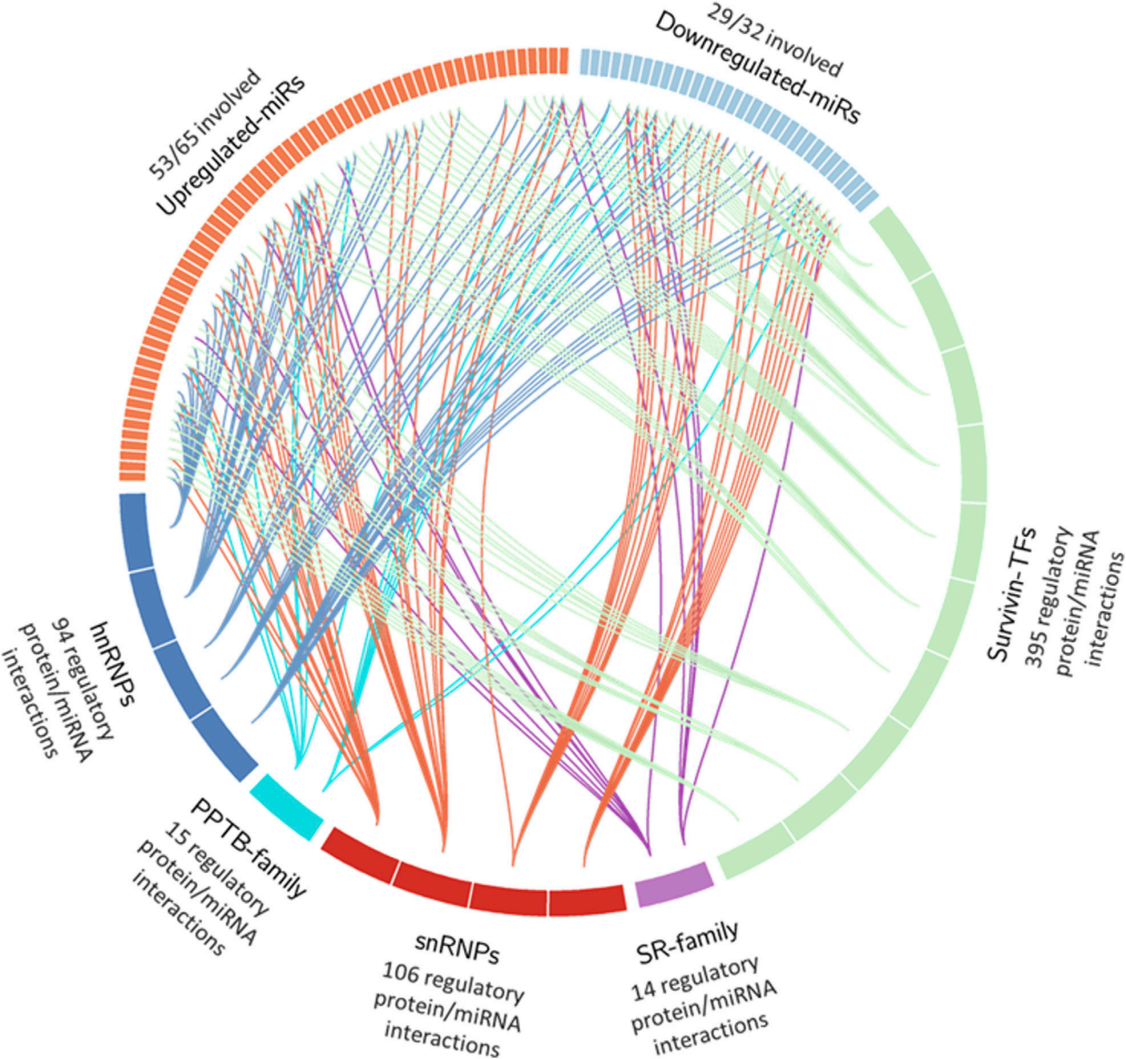
Circos plot of DEmiR interactions in UniProt and GeneHancer datasets. Proteins within the hnRNP, PPTB, snRNP, and SR families were selected, along with transcription factors with binding sites on birc5 promoter/enhancer regions. These factors were compiled into lists and loaded into the IPA target prediction tool. Interactions that were experimentally observed or predicted with high confidence in humans were selected. A circos plot was created to show all interactions between upregulated and downregulated DEmiRs. Individual sections in upregulated and downregulated DEmiRs represent individual miRNAs. Protein factors were sorted in alphabetical order and grouped into segments representing portions of the total for increased clarity of the graph.

To summarize these findings, we generated a circos plot (Figure 5). Of the 97 DEmiRs, 82 (84%) were found to interact with one or more of these families. There were a total of 395 miRNA interactions with survivin promoter or enhancer binding factors, 14 miRNA interactions in the SR family, 106 miRNA interactions in snRNPs, 15 miRNA interactions in the PPTB family, and 94 miRNA interactions in hnRNPs (Table 5). This indicates that most DEmiRs are anticipated to be involved in modulating the expression of survivin and altering alternative splicing simultaneously.

**TABLE 5:** Summary table of five protein groups assembled and represented in Figure 5.

Family	Number of miRNAs	Directional change and fold change (log2FC)			Number of protein/miRNA interactions
Survivin promotor/enhancer binding factors	71 miRNAs	46 up	24 down	−3.428 to 6.453	393 interactions
Serine-arginine-rich splicing factors	13 miRNAs	8 up	5 down	−3.428 to 3.492	14 interactions
snRNPs	48 miRNAs	29 up	19 down	−3.428 to 6.453	104 interactions
Polypyrimidine tract binding proteins	14 miRNAs	32 up	19 down	−2.982 to 3.887	92 interactions
hnRNPs	51 miRNAs	7 up	7 down	−3.428 to 6.453	15 interactions

The GeneHancer database was used to pull transcription factors that bind to survivin promoter or enhancer regions in the genome. The remaining groups make up the spliceosome and were selected using UniProt searching for the family names. IPA was used to predict targets in each of the represented proteins. The total number of interactions include miRNAs with multiple targets within the given groups.

### Comparing miEAA results with other cancer types

**FIGURE 6:**
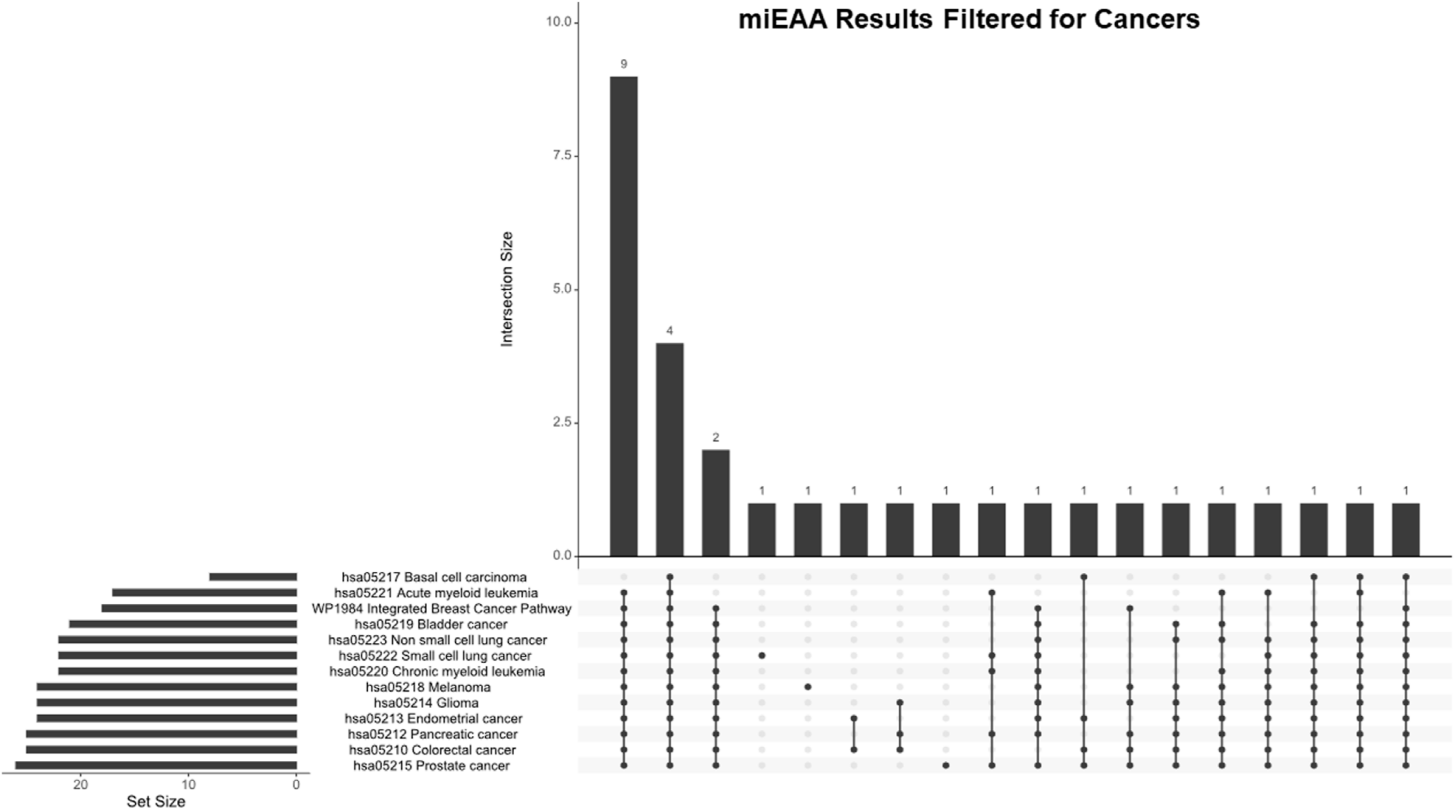
miEAA results filtered for cancer annotations. Pathways generated from the miEAA report were screened for involvement in any cancer type. All cancers found are represented. Bars indicate the number of miRNAs shared across the connected dots, as seen below. The set size represents the total number of miRNAs from our dataset that are involved in the noted pathway.

While PDAC expresses a robust chemoresistance potential, we applied these findings to many other cancer types through miEAA to determine overlaps with other cancers and chemoresistance. We generated a filtered list from the miEAA results that included all represented annotations for specific cancers, which resulted in 13 significantly represented annotations (Table 6). We compared these cancers with an upset plot (Figure 6). All cancer types shared four DEmiRs, but after excluding basal cell carcinoma, there are a total of 13 shared DEmiRs (between 48.2% and 76.5% of set sizes) between the 12 other cancers. Interestingly, pancreatic cancer shared 23 DEmiRs (92% of the set size) with colorectal cancer, 24 DEmiRs with glioma (96%), and 24 DEmiRs with prostate cancer (96% of the set size). This indicates that the chemoresistance profile generated in PDAC cell lines has differential expression in key miRNAs that are also shared in these cancer types.

**TABLE 6:** miEAA miRWalk pathways: Highlighted annotations for pancreatic cancer.

Annotation	Enrichment	*p*-value	*p*-adj-value	miRNAs
WP2256 Integrated pancreatic cancer pathway	Enriched	3.83E−04	0.0152802	27
hsa05210 Colorectal cancer	Enriched	9.25E−04	0.0152802	25
hsa05212 Pancreatic cancer	Enriched	9.25E−04	0.0152802	25
hsa05213 Endometrial cancer	Enriched	0.001282	0.0152802	24
hsa05218 Melanoma	Enriched	0.001282	0.0152802	24
hsa05219 Bladder cancer	Enriched	8.13E−04	0.0152802	21
WP1984 Integrated breast cancer pathway	Enriched	0.0022795	0.019038	18
hsa05215 Prostate cancer	Enriched	0.003366	0.0213811	26
hsa05220 Chronic myeloid leukemia	Enriched	0.0033068	0.0213811	22
hsa05222 Small cell lung cancer	Enriched	0.0033068	0.0213811	22
hsa05223 Non-small cell lung cancer	Enriched	0.0033068	0.0213811	22
hsa05217 Basal cell carcinoma	Enriched	0.0125401	0.0439439	8
hsa05221 Acute myeloid leukemia	Enriched	0.0156614	0.0457874	17

The miEAA report generated ∼180 pathways from the DEmiRs indexed by the Wald statistic. Only enriched pathways represented by > 2 miRNA interactions and a B-H adjusted *p*-value were selected for analysis. The pathways shown were screened by involvement in cancer types. (The complete, unfiltered list of miEAA findings is available upon reasonable request).

### Profile comparison with annotated miRNAs

**FIGURE 7:**
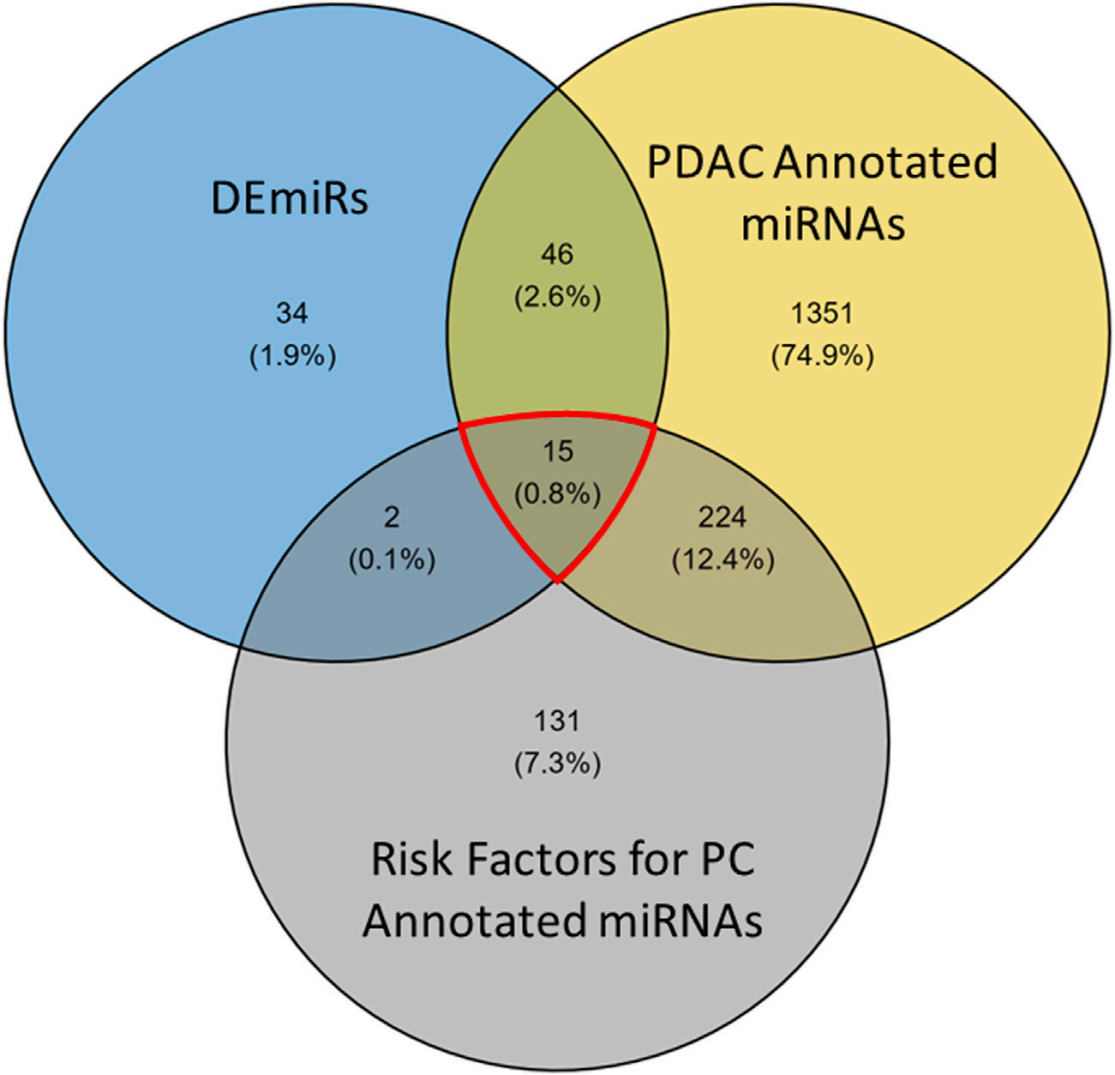
Venn diagram of miRNA factors differentially expressed in patient data and resistant cells. Risk factor and PDAC annotated miRNAs were taken from the RNADisease repository v4.0 and compared with DEmiRs between GR and GS cells to refine the 97 DEmiRs into a subset. The red highlighted region represents the 15 miRNAs used in prediction analysis.

To refine these findings into a list of the most actionable miRNAs, we filtered the DEmiRs to look at miRNAs directly associated with PDAC. The RNADisease repository V4.0 was used to collate miRNAs that are annotated for involvement in many diseases. To select for miRNAs previously reported in PDAC, we accumulated terms for keywords: “pancreatic adenocarcinoma,” “pancreatic cancer,” “pancreatic ductal adenocarcinoma,” and “pancreatic carcinoma.” This generated a list of 1,636 unique miRNAs annotated for involvement in PDAC (yellow, Figure 7). When compared to the DEmiRs (blue, Figure 7) from this study, 61/97 DEmiRs were shared between the two groups (Figure 7). To further refine this dataset, we considered risk factors (gray, Figure 7) associated with PDAC. This was done to further focus on the most aggressive factors associated with PDAC, as these risk factors have been associated with increased cancer aggressiveness and chemoresistance (Incio et al., 2016; Chen et al., 2022; Kesh et al., 2022; Vaziri-Gohar et al., 2023). To pair this with the DEmiR set, we assembled an miRNA list from the keywords: “nicotine consumption/nicotine addiction,” “diabetes,” “obesity,” “pancreatitis,” “acute pancreatitis,” “chronic pancreatitis,” and “metabolic syndrome,” The list provided 372 unique miRNAs that shared 17 factors with the DEmiR set. In conjunction, the three groups shared 15 miRNA factors (genes of interest (GOIs)), outlined in red (Figure 7).

After refining the DEmiRs to 15 key miRNAs, we compared these results to our previous findings. Of the 15 GOIs, 13 had interactions in survivin transcription factors and 12 had interactions in alternative splicing groups (Table 7). Most notably, these miRNAs are represented in the upset plots from Figures 4, 6. In the key pathways associated with cancer (Figure 4), 11 miRNAs are shared between these pathways. Four of these miRNAs are also found in the GOI set (Figure 7). When looking at 16 miRNAs shared between apoptosis and the spliceosome independently, 10 are found within the GOIs.

**TABLE 7:** 15 miRNAs found in DEmiRs, PDAC, and risk factors and their associations with survivin and alternative splicing.

miRNA	LFC	*p*-adjusted	Associations
hsa-miR-192-5p	2.844	0.0102	Survivin TFs//hnRNPs//snRNPs
hsa-miR-15a-5p	2.597	0.0123	NA
hsa-miR-29b-3p	2.581	0.0157	Survivin TFs//snRNPs//SRs
hsa-miR-20a-5p	2.239	0.0397	Survivin TFs//hnRNPs//snRNPs
hsa-miR-532-5p	2.167	9.66E−05	Survivin TFs//SRs
hsa-miR-196a-5p	2.124	0.000596	Survivin TFs//hnRNPs//PPTBs
hsa-miR-424-3p	1.906	0.00173	Survivin TFs//hnRNPs//snRNPs
hsa-miR-27b-3p	1.816	0.0372	Survivin TFs//hnRNPs//snRNPs
hsa-miR-22-3p	1.753	0.0135	Survivin TFs//snRNPs
hsa-miR-501-3p	1.708	0.00218	Survivin TFs//hnRNPs
hsa-miR-4454	1.690	0.0432	NA
hsa-miR-328-3p	1.466	0.0225	Survivin TFs//hnRNPs//snRNPs
hsa-miR-769-5p	1.312	0.0202	Survivin TFs
hsa-miR-361-3p	1.054	0.0372	Survivin TFs//hnRNPs//snRNPs//SRs//PPTBs
hsa-miR-346	2.422	0.0372	Survivin TFs//hnRNPs//snRNPs//SRs//PPTBs

LFC and *p*-adjusted represent the differential expression between GR and GS cells. Spliceosome and survivin transcription factor groups were used to integrate these 15 key miRNAs to the apoptosis inhibition and survivin alternative splicing previously seen in GR cells. SR, serine rich; TF, transcription factor; PPTB, polypyrimidine tract binding; hnRNP, heterogenous nuclear ribonucleoprotein; snRNP, small nuclear ribonucleoprotein.

### Patient data analysis

To translate these data into a more translational study, we pursued the ability of these GOIs to predict patient outcomes based on data from *The Cancer Genome Atlas* (TCGA). The PAAD and CPTAC3 datasets from the TCGA contained 183 and 179 PDAC patients, respectively, and were chosen for this study. Available metadata in these sets were incomplete. As a result, different strategies were required to link the data to our chemoresistance cell line model. We were unable to extract tumor recurrence data from the PAAD dataset; therefore, we sorted the patients into two groups based on patients who had received prior treatment *versus* patients who did not receive treatment before tumor sampling. This strategy was used to model the GOIs to distinguish between patients who had been treated with therapy and those who had not yet been treated. We sorted the patients in the CPTAC3 patient dataset into two groups, recurrent and nonrecurrent, and used these patient groups as a clinical analog to our chemoresistant/recurrent cell line model. Despite the lack of complete metadata, we were able to identify promising predictions based on the GOI set.

**FIGURE 8:**
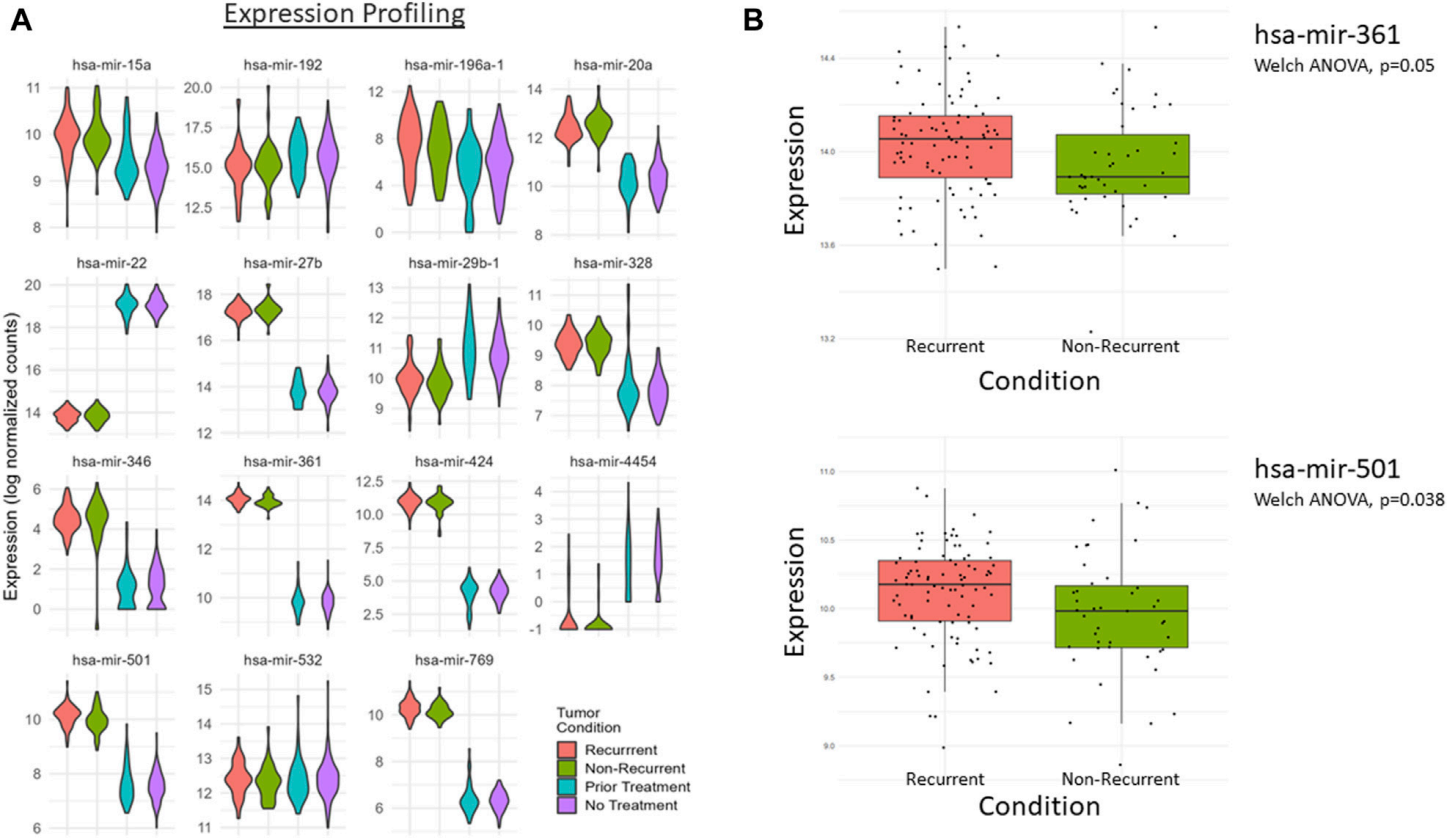
**(A)** Violin plots of the 15 key differentially expressed miRNAs from the PAAD and CPTAC3 patient datasets. CPTAC3 represents the recurrent (red) and nonrecurrent (green) groups, whereas the PAAD dataset represents the prior treatment (blue) and no prior treatment group (purple). Data are grouped based on available metadata from the CPTAC3 and PAAD datasets. **(B)** A significant difference was measured between recurrent and nonrecurrent tumor conditions for hsa-mir-501 and hsa-mir-361, *p-value <0.05.

**FIGURE 9:**
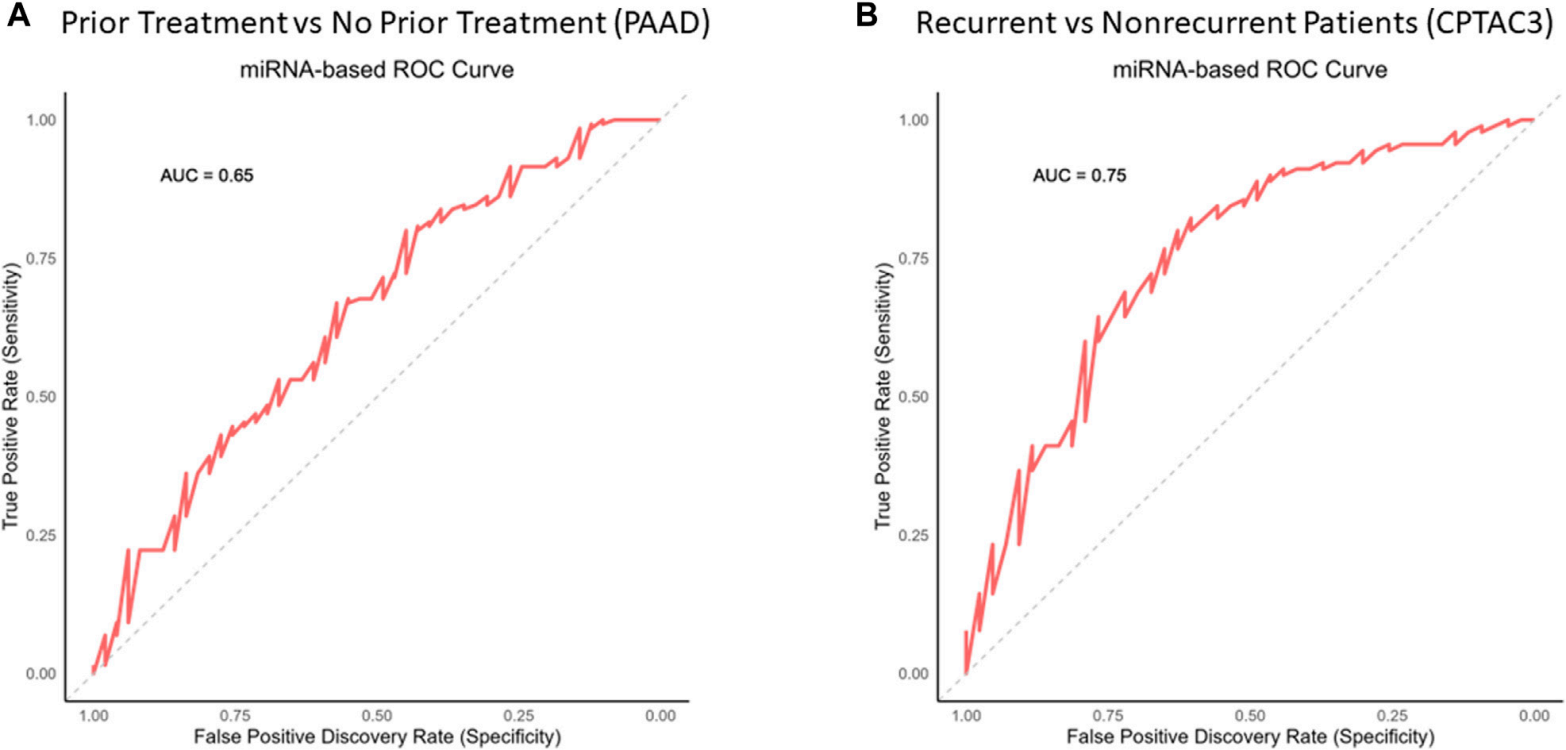
Prediction analysis via the ROC curve on key miRNAs. The total GOI set of 15 miRNAs was used to generate a prediction report via logical regression and ROC analysis. The red line represents the running threshold “tradeoff” of specificity and sensitivity. The gray line represents a no-benefit prediction model to give context. The area under the curve (AUC) is the overall accuracy potential for these miRNAs to distinguish between **(A)** prior treatment and no prior treatment from the PAAD patient group and **(B)** recurrent and nonrecurrent tumors from the CPTAC3 patient group.

The expressions of each of these miRNAs in the GOI set between the patient groups (Table 8) are summarized by a violin plot in Figure 8A. Only a few of these miRNAs were significantly differentially expressed between corresponding groups. There were no significant differences between the mean expression of prior treatment vs. no treatment from TCGA-PAAD. However, two were significantly differentially expressed in recurrent vs. nonrecurrent tumors, hsa-mir-501 and hsa-mir-361 (Figure 8B). While the mean expression of each group lacked much significance, individual patients expressed enough variance of one or more of these factors to provide a suitable specificity and sensitivity for prediction (Figure 9). When utilizing the total set of 15 GOIs in a logical regression-modeled ROC analysis, we could distinguish with a modest threshold the belonging to their respective groups. The AUC was 0.65 and 0.75 in the TCGA-PAAD and CPTAC3 groups, respectively (Figures 9A, B). The AUC represents the overall accuracy of correctly sorting the groups based on these miRNAs. This predictive model could sort patients into their correct groupings with reasonable accuracy. We anticipate that additional refinements of these selected miRNAs in other datasets will continue to improve the ability to distinguish between tumors that progress after chemotherapy.

**TABLE 8:** Patient numbers and subgroups used for differential miRNA expression in pancreatic adenocarcinoma.

CPTAC3 (recurrent vs. nonrecurrent data)		TCGA-PAAD (treatment vs. no treatment)	
Nonrecurrent	N = 43	No treatment	N = 49
Recurrent	N = 90	Treatment	N = 130
Total patients	N = 133	Total patients	N = 179

## Discussion

Pancreatic ductal adenocarcinoma (PDAC) is in urgent need of improved treatment strategies. PDAC is particularly lethal because most patients will experience recurrence within 2–5 years of their initial diagnosis (Copur et al., 2020). Recurrence is attributed to the ineffectiveness of both gemcitabine and FOLFIRINOX. Chemoresistance to these drugs is not only common but rapidly developed within a pancreatic tumor. In order to develop a more efficacious and targeted therapy, we sought to identify key miRNA factors and target proteins that are differentially expressed in response to therapeutic induction.

In this study, we found novel mechanisms and insights on chemoresistance development in two variably resistant cell lines. MIA-PaCa-2 parental cells are sensitive to gemcitabine, as opposed to the highly resistant MIA-PaCa-2 GR cells developed in this lab. Previous research found that the MIA-PaCa-2 GR cells were significantly resistant to the full therapeutic regimen of FOLFIRINOX and gemcitabine (Fuller et al., 2022). To address the survival and resistance we observed, we conducted miRNA-seq and identified key expression changes that promote chemotherapy resistance in the GR cells. miRNAs were selected because of their ability to post-transcriptionally regulate gene expression by binding to the 3’ UTR of a target mRNA, leading to mRNA degradation or suppression of translation (Guo et al., 2020). In essence, miRNA expression can be seen in the same light as gain of function mutations (oncogene) or loss of function mutations (tumor suppressor genes) and thus serve important roles in apoptosis, invasion and metastasis, proliferation, and resistance (Chen, 2005; Yonemori et al., 2017; Guo et al., 2018).

This study focuses on the comprehensive bioinformatic analysis of miRNA profiles connecting protein target prediction and represented pathway predictions in pancreatic cancer to define mechanisms that promote the transition from chemosensitive to chemoresistant cells. Using NGS, we evaluated differentially expressed miRNAs in the Gem-resistant MIA PaCa-2 cell line compared to its Gem-sensitive parent cell line and discovered 97 significant (q-value < 0.05), differentially expressed miRNAs out of a library of 1867 total sequenced miRNAs. This dataset was subsequently used for gene ontology analysis and pathway prediction.

Among differentially expressed miRNAs, the expressions of miR-935, miR-4668-5p, and miR-4271 were downregulated, while the expressions of miR-34a-5p, miR-3529-5p, and miR-205-5p were upregulated in association with GR. A similar pattern of downregulation of miR-935 was noted in non-small-cell lung cancer (NSCLC), which resulted in increased sensitivity to paclitaxel (Peng et al., 2018). MiR-205-5p has been linked to cell proliferation and heightened drug responsiveness in basal cell carcinoma (BCC), squamous cell carcinoma (SCC), and melanoma. However, unlike in our investigation, where its influence is anticipated to stem from interactions with the multidrug resistance-associated protein family, miR-205-5p′s suppressive impact in skin cancer is attributed to its targeting of members within the tumor necrosis factor-α family (Ge et al., 2021).

In addition, miRNAs miR-663, miR-10a-5p, miR-34a-5p, and miR-205-5p appear to be indirectly associated with regulating drug efflux through the breast cancer resistance protein (BCRP/ABCG2), ABCC10, and multidrug resistance-associated protein 2 (MDR2) as a result of GR in the MIA PaCa-2 cell line. MiR-663, when hypomethylated, has been shown to induce chemoresistance in breast cancer cells by targeting heparin sulfate proteoglycan 2 (HSPG2). Its association with chemoresistance may occur through its ability to modulate growth factor signaling pathways, protect cancer cells from apoptosis, and alter the tumor microenvironment to promote survival (Hu et al., 2013). Furthermore, miR-663 has also been shown to be overexpressed in Taxol-resistant ovarian cancer cells and has been characterized as a significant prognosis marker in chemoresistance patients (Kim et al., 2014). MiR-10a increases cisplatin resistance in lung adenocarcinoma circulating tumor cells by targeting the PI3K/Akt pathway (Huang et al., 2020) and also targets TFAP2C to promote Gem resistance in PDAC (Xiong et al., 2018). In this same study, TFAP2C expression decreased the migration and invasion capability of PDAC cells. In this way, miR-10a acts as an oncogene to promote metastatic behavior as well as Gem resistance in PDAC cells. MiR-34a and miR-205-5p have both attracted extensive interest due to their involvement in many different cancer types. MiR-34a inhibited the migration, invasion, and proliferation (Avtanski et al., 2016) and modulated drug sensitivity (Kastl et al., 2012; Li et al., 2012) in breast cancer cells by affecting antiapoptotic genes Bcl-2 and CCND1 (Avtanski et al., 2016) and the Ras family proteins, NOTCH1 and PRKD1 (Li et al., 2012).

By conducting gene ontology analysis using IPA and miEAA, we discovered that these miRNAs were significantly represented in major cancer pathways, as seen in Figures 3A, B, and 4. Several cancer-promoting pathways are significantly represented in these figures. Additionally, we observed potential crosstalk between other cancer diseases, as shown in Figure 6. Interestingly, immunological and inflammatory pathways were also significantly represented (Figure 3A). PDAC initiation and inflammation are closely related, as the risk factors for PDAC, such as smoking, diabetes, obesity, high-fat diet, and alcoholism, promote inflammatory signaling through the release of IL-6 and NF-κB. As we are comparing resistance to sensitivity, the DEmiRs involved in inflammatory pathways may also suggest that the maladies promoting inflammation may potentiate the onset of chemoresistance. This is also evidenced by the abundant overlap in miRNAs associated with risk factors for PDAC development (Figure 7).

It is important to note that throughout the gene ontology studies conducted, there is no indication of whether these effects are positive or negative to the overall pathway. Further studies are required to define the exact effect that the DEmiRs play in these pathways. However, these data highlight the intersection points of DEmiRs in similar pathways, which gives insights to generate future studies. Additionally, miRNA annotations from GO and miEAA give a starting point for a refined list of predictive/prognostic biomarkers (Figure 9). In all, the future directions for this study are to refine this predictive dataset to improve its sensitivity and specificity, gather a more robust patient sample, and translate this miRNA dataset to target chemoresistance-promoting pathways in PDAC.

We observed important molecular interactions, particularly with alternative splicing and apoptosis induction. Previous studies with these resistant cells indicated that survivin alternative splicing, especially the overexpression of the survivin 2β isoform, significantly increased chemoresistance to Gem and FOLFIRINOX (Fuller et al., 2022). Because miRNAs play a role in both promoting and participating in alternative splicing, they may serve as a crucial regulatory mechanism to evade apoptosis. As seen in the circos plot interactions (Figure 5; Table 5), 84.5% of all DEmiRs (82/97) are predicted to be involved in survivin gene transcription, alternative splicing machinery, or both. This much overlap in the profile provides significant preliminary data to explore the interactions with alternative splicing as a potential therapeutic target to combat chemoresistance.

In addition, it is also evident that several of these miRNAs are expressed among patients with chemoresistant tumors. In both the PAAD and CPTAC3 datasets, we were able to distinguish between the groups with reasonable accuracy through ROC analysis. The AUC in Figure 9 represents the overall accuracy (sensitivity/specificity tradeoff) in distinguishing and sorting patients into their respective groups. These 15 miRNAs in the GOI set between the PAAD and CPTAC3 patients indicate their likely roles in aggressiveness or response to therapeutics. The lack of consistent significance between expression averages in each group suggests that each patient may only differentially express a handful of these miRNAs in response to therapy or recurrence. Generalized ROC interpretation estimates that models with an AUC <0.7 indicate acceptable discrimination between groups (Mandrekar, 2010). However, general thresholds are not often correct measures for acceptable sensitivity and specificity tradeoffs. It is vital to address cost, risk, and benefit to determine the acceptable thresholds of false positivity and false negativity. In this experiment, we determined that this model is an acceptable baseline, but the model requires refinement before it can be implemented clinically. Furthermore, the lack of available data in the TCGA to conduct an miRNA expression analysis on mature miRNA sequences significantly impacts this study. We plan on further improving this predictive model in a future study utilizing PDAC patients.

In line with these observations, miRNAs from this dataset are overrepresented within the data shown in Figure 5. Table 7 highlights the represented pathways between the GOIs, spliceosome factors, and survivin transcription factors. Interestingly, 13/15 GOIs are involved in one or more of these pathways: 13 interact with survivin transcription factors alone and 12 interact with the splicing factors alone. Seven miRNAs are common to all groups of the spliceosome, survivin transcription factors, and GOIs. This evidence indicates consistency between the miRNA groups and is most probably the major reason for the predictive capacity seen in the patients. These findings reinforce the extrapolation of these factors promoting chemoresistance and provide a starting point for the continuation of this study to look at these key miRNAs as prognostic biomarkers and potential contributors to chemoresistance.

In order for this research to attain relevance and be applied to PDAC generally, several limitations need to be addressed. While there is great power in taking a sensitive cell line and cultivating chemoresistance within it to determine epigenetic modifications responsible, these miRNA modifications might not be well-conserved between other PDAC cell lines or patients. Given the limitation of utilizing TCGA data for PDAC, namely, the lack of available metadata and incomplete, immature miRNA profiles, it is difficult to draw further relevant findings from this dataset. It is, therefore, vital that more miRNA-based PDAC studies are built within additional cell lines and patients to better understand the role these miRNAs have in conveying chemoresistance.

In conclusion, we have identified a series of differentially expressed miRNAs induced by long-term Gem exposure leading to acquired resistance. Further study of this miRNA signature will need to be conducted in a patient cohort who has already become resistant to Gem and/or FOLFIRINOX, as well as in a longitudinal study of newly diagnosed PDAC patients following their disease etiology. This will enable a biomarker comparison that directly measures the miRNA changes that occur as patients develop reduced sensitivity and eventual resistance and allows direct comparison to those patients who have already become resistant to Gem and/or FOLFIRINOX.

## Scope statement

The manuscript explores the role of microRNAs (miRNAs) in gemcitabine (Gem) resistance in pancreatic adenocarcinoma (PDAC), a major clinical challenge. By analyzing miRNA expression profiles in Gem-sensitive and Gem-resistant PDAC cell lines, the study identifies 97 differentially expressed miRNAs associated with chemoresistance. These miRNAs are implicated in critical biological processes such as cell proliferation, migration, chemo-sensitization, apoptosis, and angiogenesis. Notably, the research extends beyond cell lines to analyze patient samples, offering potential clinical or translational relevance. The study’s approach aligns with the journal’s specialty section on “Next-Generation Sequencing (NGS) and Cancer: New Steps Towards Personalized Medicine” by leveraging next-generation sequencing and analytical software to uncover regulatory miRNAs involved in chemoresistance pathways. The findings provide insights into potential therapeutic targets and diagnostic markers for PDAC with Gem resistance, contributing to the advancement of precision oncology and personalized treatment strategies.

## Data Availability

The original contributions presented in the study are publicly available. This data can be found here: Gene Expression Omnibus (GEO) repository, accession number: GSE269190.
