# Supplementary figures and images for: miRNA signatures underlie chemoresistance in the gemcitabine-resistant pancreatic ductal adenocarcinoma cell line MIA PaCa-2 GR
Source: Front Genet. 2024 Jun 11;15:1393353. doi: 10.3389/fgene.2024.1393353 (PMC11196613; doi:10.3389/fgene.2024.1393353)

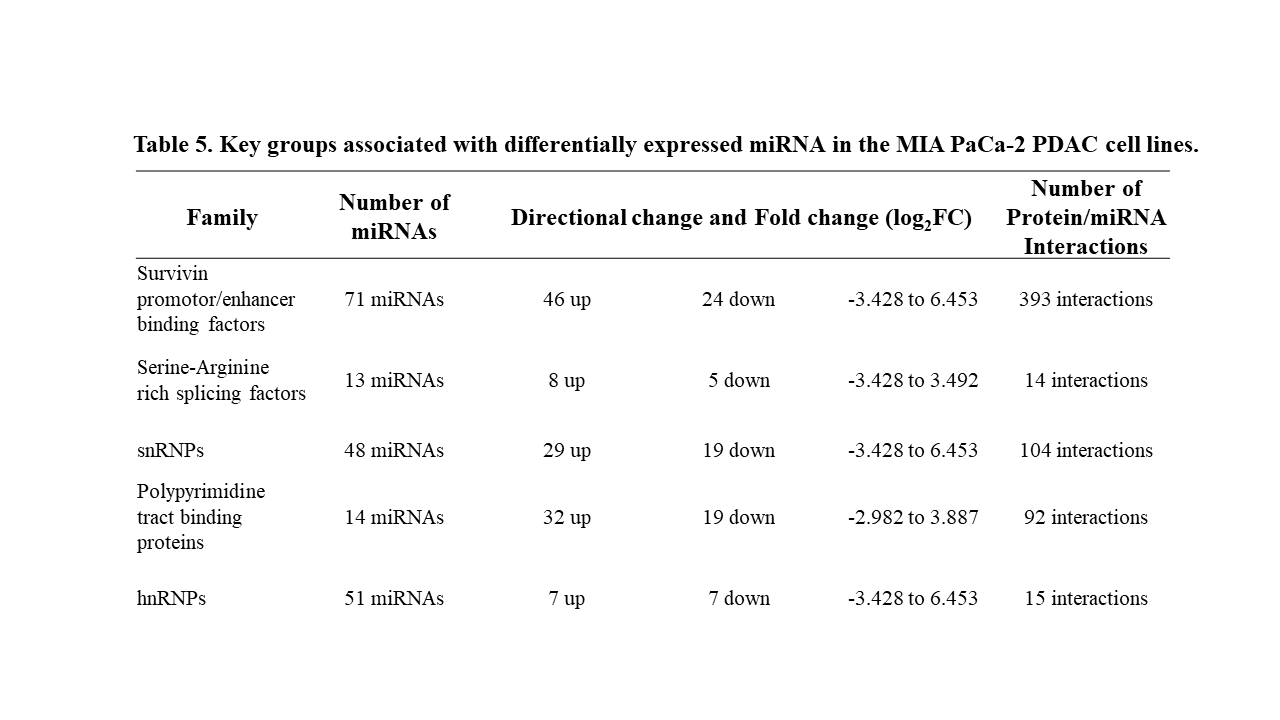

Supplement: Supplementary file 1 [file Image6.TIF]

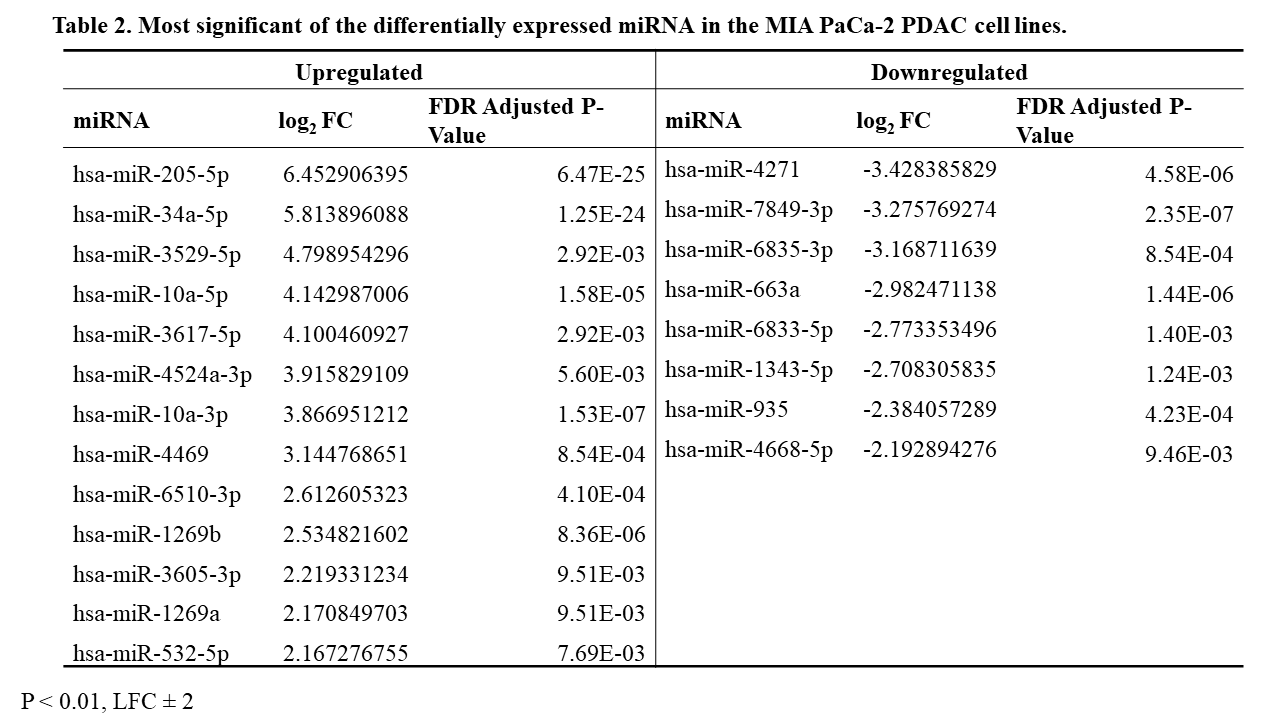

Supplement: Supplementary file 2 [file Image3.TIF]

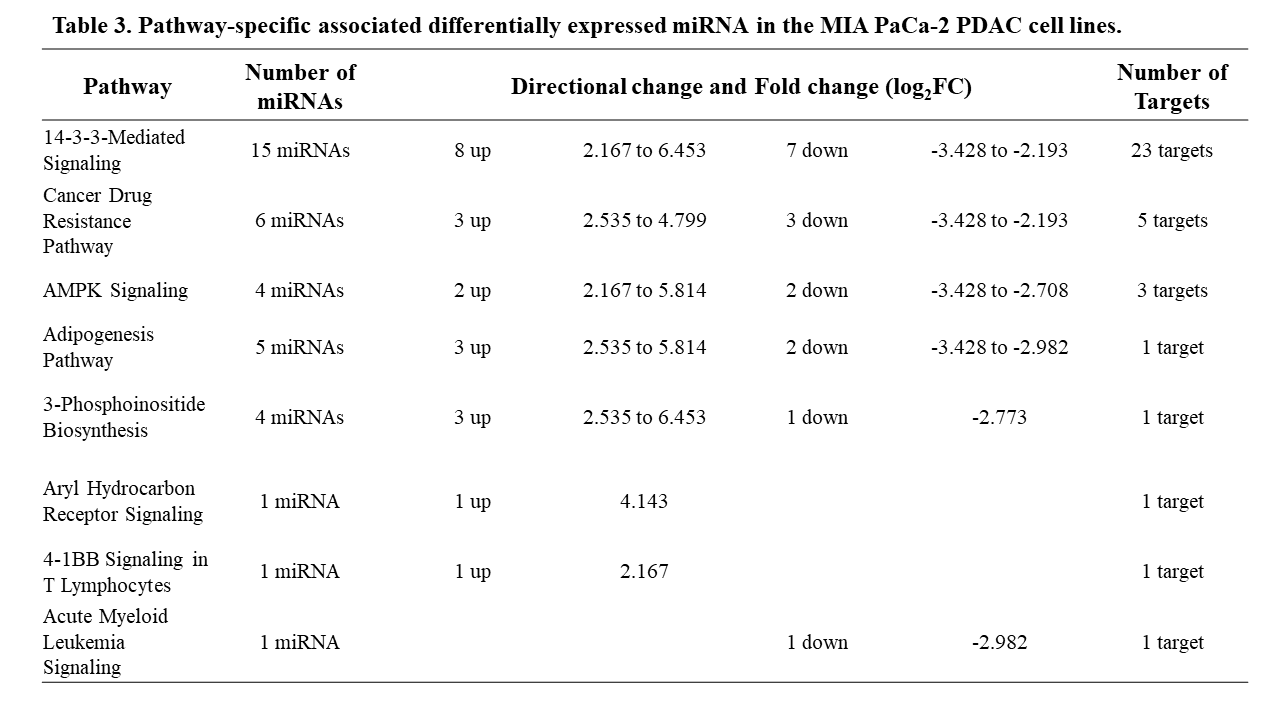

Supplement: Supplementary file 3 [file Image4.TIF]

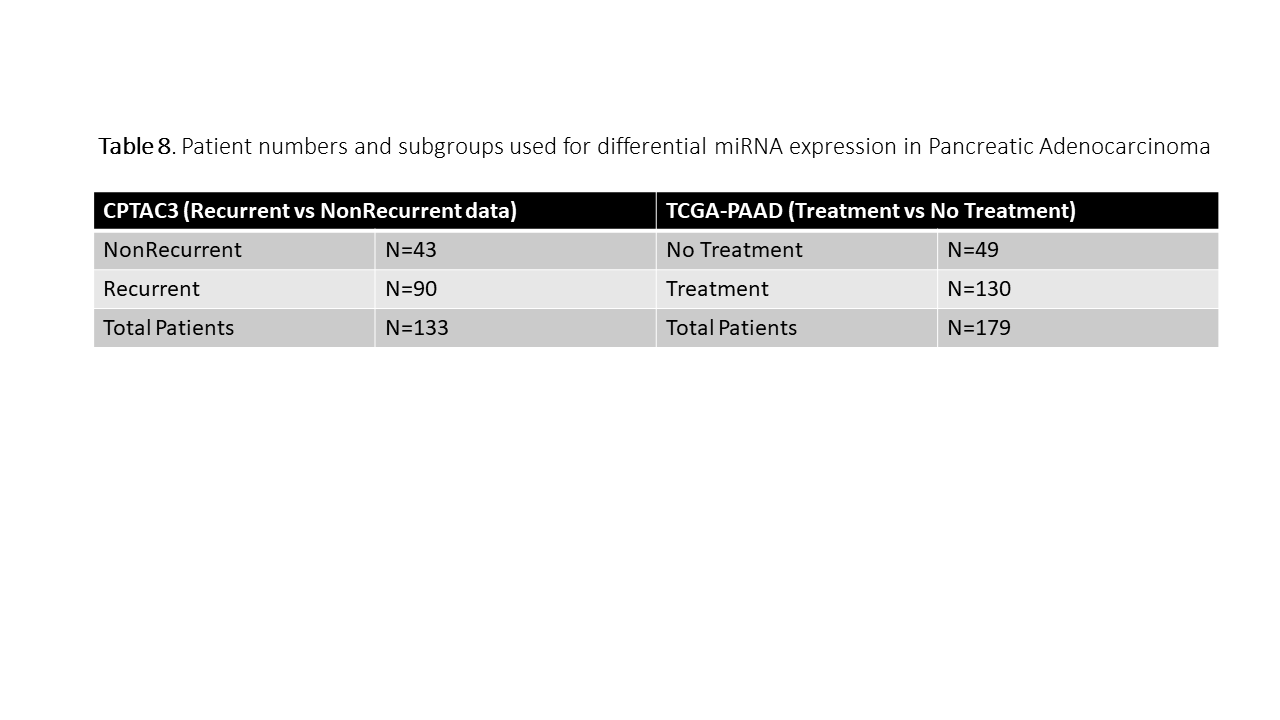

Supplement: Supplementary file 4 [file Image9.TIF]

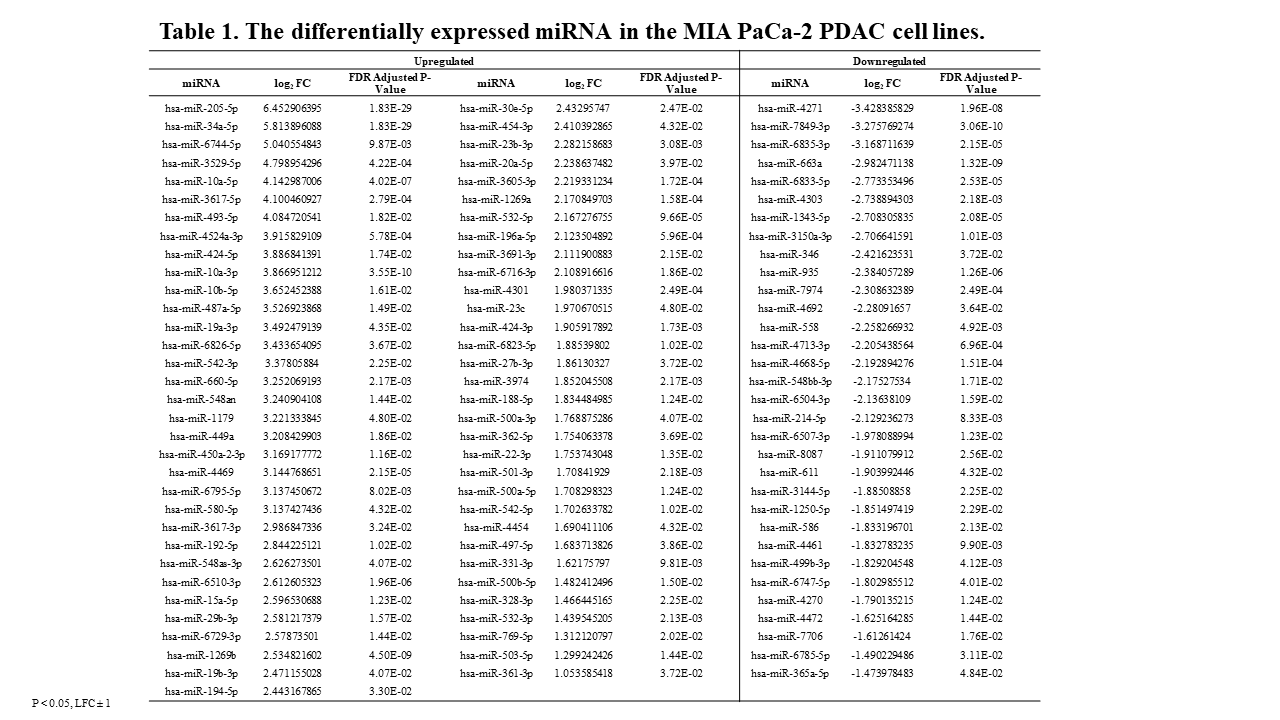

Supplement: Supplementary file 5 [file Image2.TIF]

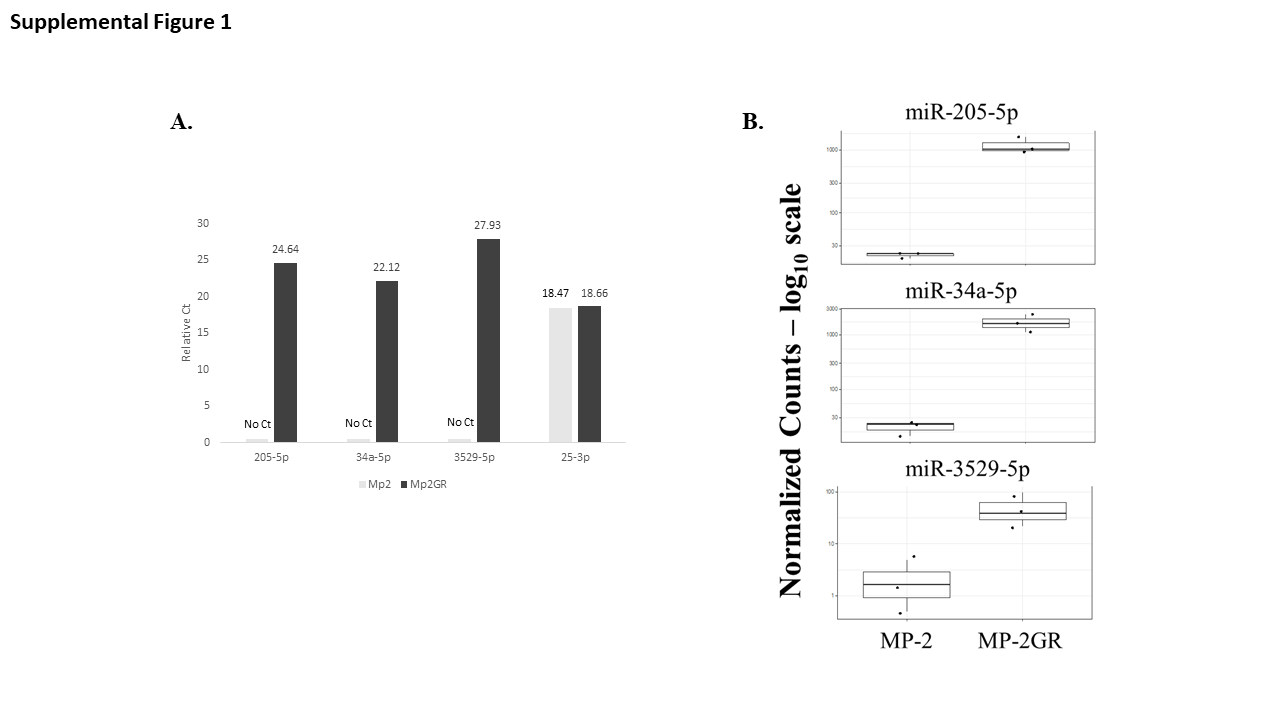

Supplement: Supplementary file 6 [file Image1.TIF]

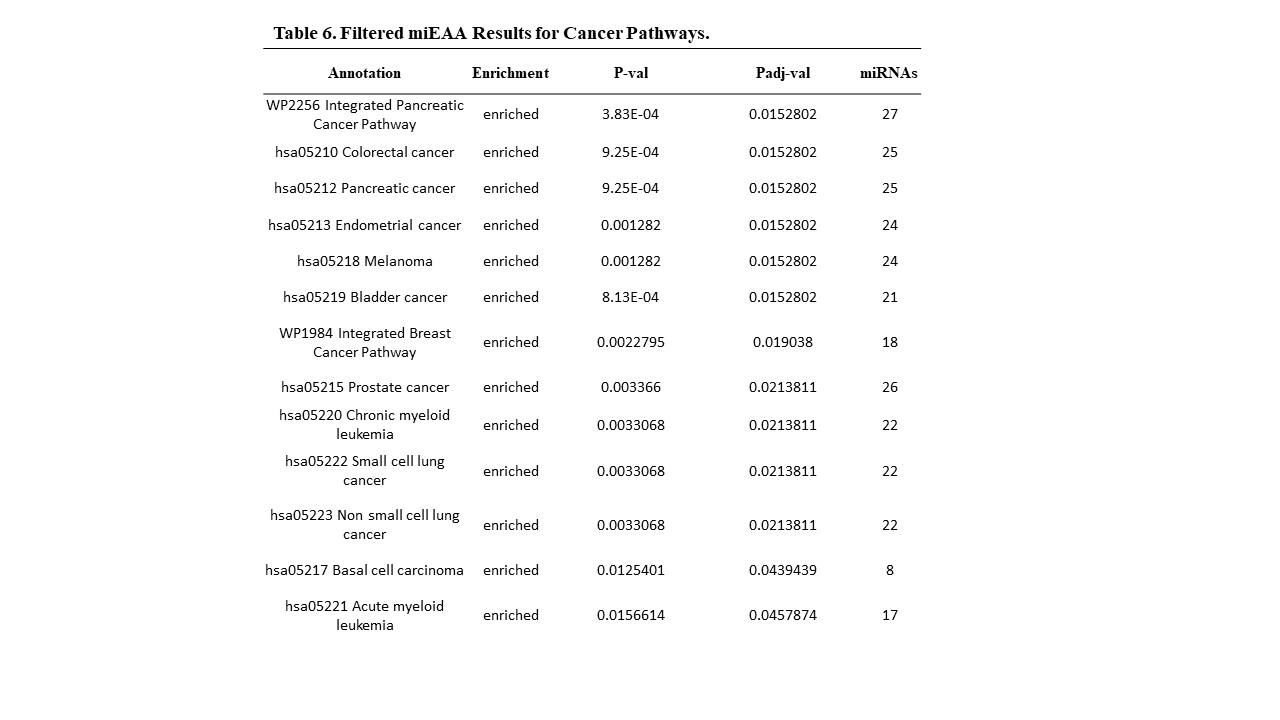

Supplement: Supplementary file 7 [file Image7.TIF]

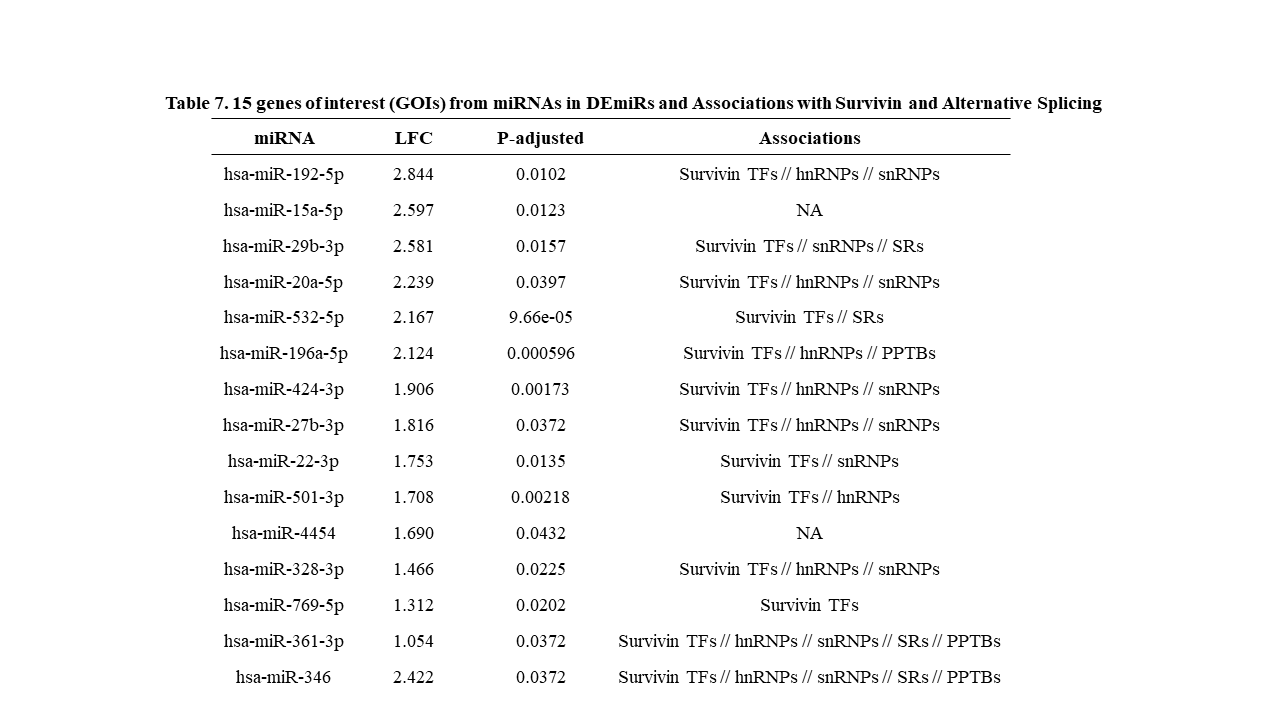

Supplement: Supplementary file 8 [file Image8.TIF]

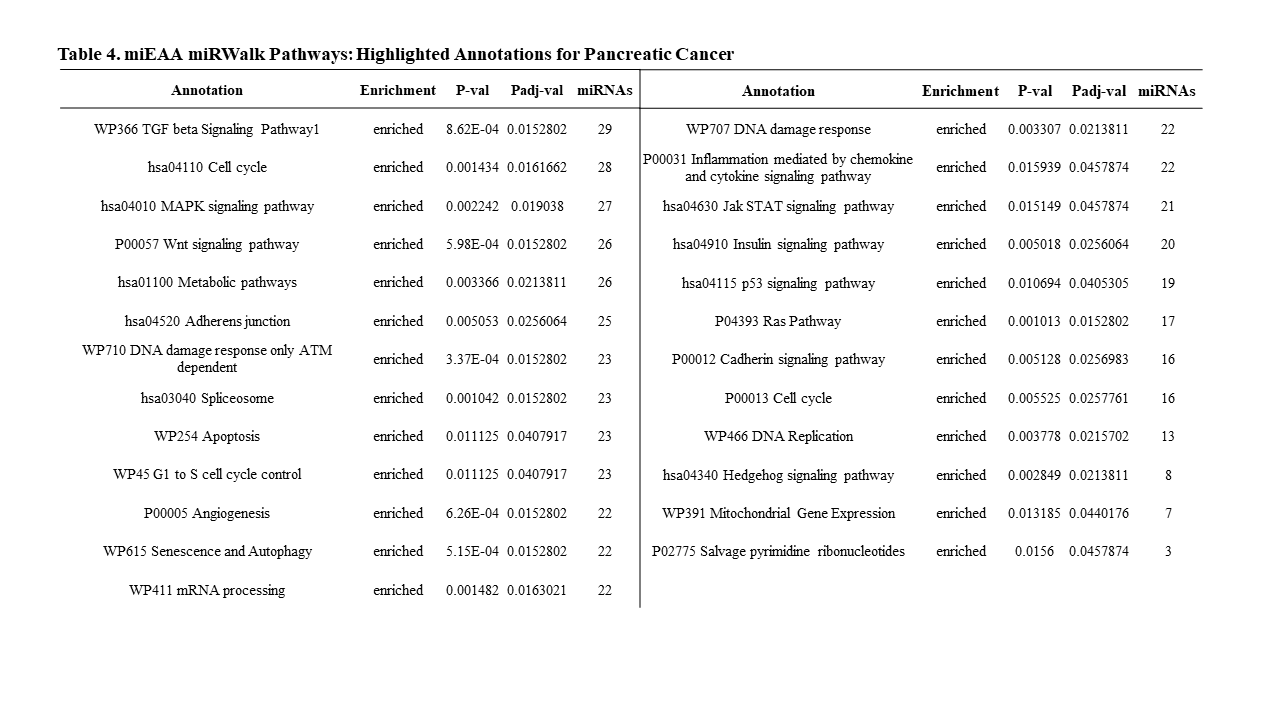

Supplement: Supplementary file 9 [file Image5.TIF]
